# Lilrb4a Suppression Reprograms Microglia to Mitigate APOE4‐Associated Amyloid Plaques and Cerebral Amyloid Angiopathy in Association With a PPAR‐Linked Pro‐Clearance State

**DOI:** 10.1002/advs.202524167

**Published:** 2026-06-22

**Authors:** Changxu Nie, Ruixi Yang, Xiaotong Wang, Ping Jia, Xueqi Zhang, Yaqi Dai, Xue Bai, Sijia Duan, Yufeng Li, Peng Zheng, Xin Tian, Li Jiang, Chao Wang

**Affiliations:** ^1^ Department of Psychiatry The First Affiliated Hospital of Chongqing Medical University Department of Neurobiology School of Basic Medical Sciences Key Laboratory of Major Brain Disease and Aging Research (Ministry of Education) Chongqing Medical University Chongqing China; ^2^ Guangzhou National Laboratory Graduate School of Guangzhou Medical University Guangzhou Guangdong China; ^3^ Department of Neurology The First Affiliated Hospital of Chongqing Medical University Jinfeng Laboratory Chongqing China; ^4^ Department of Geriatrics Laboratory of Research and Translation for Geriatric Diseases Department of Neurology The First Affiliated Hospital of Chongqing Medical University The First Affiliated Hospital of Chongqing Medical University Chongqing Key Laboratory of Major Neurological and Mental Disorders Key Laboratory of Major Brain Disease and Aging Research (Ministry of Education) Chongqing Medical University Chongqing China; ^5^ Department of Neurosurgery The First Affiliated Hospital of Chongqing Medical University Key Laboratory of Major Brain Disease and Aging Research (Ministry of Education) Chongqing Medical University Chongqing China

**Keywords:** amyloid plaque, APOE4, cerebral amyloid angiopathy, *LILRB4*, *Lilrb4a*, microglia, PPAR‑γ

## Abstract

The mouse gene *Lilrb4a*, an ortholog of human leukocyte immunoglobulin‐like receptor B4 (*LILRB4*), is markedly upregulated in microglia in Alzheimer's disease models and has been implicated in Apolipoprotein E (APOE)‐related signaling. However, its contribution to amyloid pathology under an APOE4 background remains unclear. Here, 5xFAD mice carrying human *APOE4* were used to assess the impact of *Lilrb4a* reduction by genetic deletion or antisense oligonucleotide treatment. Both approaches significantly reduced cortical amyloid plaque burden and APOE4‐associated cerebral amyloid angiopathy without altering amyloid‐β (Aβ) production. Bulk RNA sequencing identified enrichment of peroxisome proliferator‐activated receptor (PPAR)‐related and broader metabolic pathways in *Lilrb4a*‐deficient mice. Consistently, biochemical analyses showed reduced p‐SHP‐2, NF‐κB‐p65, and p‐STAT1, increased p‐STAT3, and induction of anti‐inflammatory and clearance‐associated effectors, including Arg‐1, TGF‐β, and Cyp2e1. In primary microglia, pharmacological interrogation supported a functional contribution of PPAR‐γ signaling to the enhanced Aβ uptake and degradation associated with *Lilrb4a* suppression, whereas PPAR‐γ agonism recapitulated key pro‐clearance phenotypes in vitro and attenuated amyloid pathology in vivo. Together, these data support Lilrb4a as an APOE4‐associated microglial checkpoint candidate linked to impaired amyloid clearance and identify a PPAR‐linked pro‐clearance program as a potential downstream component of this response.

## Graphical abstract

Targeting *Lilrb4a* in Apolipoprotein E4 (APOE4)‐associated Alzheimer's disease (AD) reprograms microglia toward a beneficial, phagocytic state. Genetic deletion or antisense inhibition of *Lilrb4a* suppresses p‐SHP2/NF‐κB/STAT1 signaling, restores PPAR‐linked lipid and energy metabolism, and reduces amyloid plaque burden and cerebral amyloid angiopathy, ultimately improving microglial function and cognitive performance in APOE4‐driven AD mouse models.

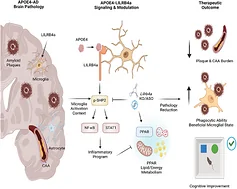

## 1 Introduction

Alzheimer's disease (AD) is the leading cause of dementia, marked by progressive cognitive decline and by characteristic lesions such as extracellular β‐amyloid (Aβ) plaques, cerebral amyloid angiopathy (CAA), and intracellular neurofibrillary tangles formed by hyperphosphorylated tau [1, 2, 3]. Among genetic risk factors, the *Apolipoprotein E4* (*APOE4*) allele exerts the strongest effect on risk for late‐onset AD, promoting greater amyloid accumulation, earlier disease onset, and more pronounced cerebrovascular dysfunction than the *APOE3* isoform [4, 5, 6]. These clinical observations underscore the need to define molecular checkpoints by which APOE4 shapes innate immune responses in the brain.

Microglia, the resident immune cells of the central nervous system, are essential modulators of AD pathology [7, 8, 9]. In early disease stages, microglia can limit amyloid burden by phagocytosing Aβ aggregates; however, under chronic activation—particularly in *APOE4* carriers—microglia shift toward a pro‐inflammatory phenotype that reduces phagocytic capacity and potentiates synaptic and vascular injury [10, 11, 12]. Restoring microglial clearance capacity while rebalancing inflammatory tone has therefore emerged as an important therapeutic concept in AD research.

Leukocyte immunoglobulin‐like receptor B4 (LILRB4) is an inhibitory immune receptor expressed in myeloid cells and markedly upregulated in microglia of human AD brains and has been regarded as a marker of disease‐associated microglia [13, 14, 15, 16]. In mice, the *Lilrb4* locus encodes two isoforms, *Lilrb4a* and *Lilrb4b*, with *Lilrb4a* being the dominant brain‐expressed variant [15, 17]. *Lilrb4a* contains cytoplasmic immunoreceptor tyrosine‐based inhibitory motifs that recruit Src homology 2 domain‐containing protein tyrosine phosphatases (SHP‐2), thereby dampening activation pathways such as nuclear factor kappa‐B (NF‐κB) signaling [18, 19]. Emerging evidence indicates that LILRB4/Lilrb4a intersects with APOE‐related signaling in microglial contexts, positioning Lilrb4a as a potential APOE4‐associated inhibitory checkpoint that may shape downstream inflammatory responses, lipid‐handling pathways, and metabolic remodeling programs, particularly within plaque‐associated microglial states [14, 15]. SHP2 activation via *Lilrb4a* engagement may, in turn, modulate NF‐κB and Janus kinase‐signal transducer and activator of transcription (JAK–STAT) axes to suppress peroxisome proliferator‐activated receptorγ (PPAR‑γ), a transcriptional regulator closely linked to lipid metabolism, anti‐inflammatory tone, and sustained phagocytosis.

Nevertheless, three key questions remain unresolved: (i) whether *Lilrb4a* directly contributes to amyloid plaque and CAA development in vivo under *APOE4* background; (ii) whether downregulating *Lilrb4a* can reprogram microglial inflammatory and metabolic pathways toward enhanced phagocytosis and improved clearance of amyloid, independent of Aβ production; (iii) whether PPAR‑γ‐linked programs participate downstream of *Lilrb4a* modulation and whether pharmacological activation can phenocopy selected aspects of the clearance phenotype.

To address these gaps, we used 5xFAD mice carrying human APOE4 (5EL) and reduced *Lilrb4a* expression via either genetic knockout or in vivo antisense oligonucleotide (ASO)‐mediated perturbation. We coupled quantitative histopathology of plaques and CAA with bulk RNA sequencing to identify transcriptional shifts, and functional assays in *BV2* and primary microglia to define effects on Aβ uptake and digestion. In parallel, we tested the in vivo impact of selective PPAR‑γ agonism on amyloid pathology. Our data support a model in which *Lilrb4a* suppression promotes a pro‐clearance microglial state and ameliorates amyloid pathology in this *APOE4*‐associated setting.

## 2 Results

### 2.1 LILRB4 Shows Stronger APOE4‐Associated Co‐IP Signals in the Tested Contexts and is Upregulated in APOE4 and Amyloid‐Related Settings

**FIGURE 1:**
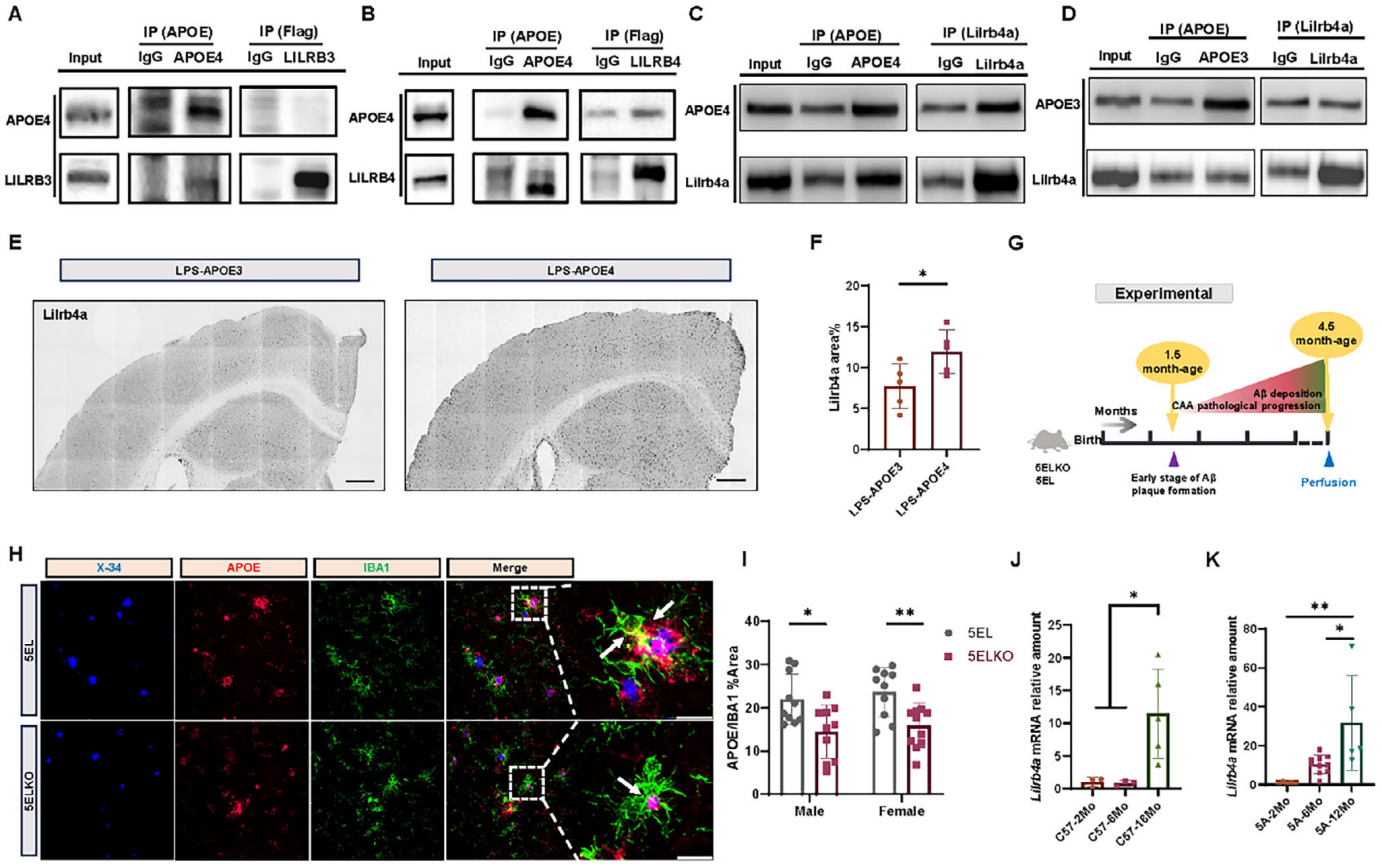
LILRB4 shows stronger APOE4‐associated Co‐IP signals in the tested contexts and is upregulated in APOE4 and amyloid‐related settings. (A,B) Reciprocal Co‐IP of human APOE4 with Flag‐tagged human LILRB3 (A) or Flag‐tagged human LILRB4 (B) in HEK293T cells. Schematic representation of Co‐IP between LILRB3/LILRB4 and APOE4 protein. Immunoprecipitation with anti‐APOE or anti‐Flag antibodies, followed by immunoblotting for APOE4 and LILRB3/LILRB4. Input lysates are shown as positive controls, and IgG immunoprecipitation serves as a negative control; (C, D) Reciprocal Co‐IP of endogenous APOE and LILRB4 in microglia isolated from 12‐month‐old APOE4 (C) or APOE3 (D) mice 24 h after intraperitoneal LPS injection. Cell lysates were immunoprecipitated with anti‐APOE or anti‐LILRB4 antibodies and immunoblotted as indicated. Input lysates are shown as positive controls, and IgG immunoprecipitation serves as a negative control; (E) Representative Lilrb4a immunostaining in cortical sections from LPS‐treated APOE3 and APOE4 mice (10×, scale bar = 500 µm); (F) Quantification of Lilrb4a‐positive area in the cortex (*n* = 4–5); (G) Schematic diagram of the experimental design; (H) Representative confocal images of X‐34 (blue), APOE (red), and IBA1 (green) staining in cortical sections from 5EL and 5ELKO mice; arrows indicate plaque‐associated microglia containing APOE signal (60× oil, scale bar = 50 µm); (I) Quantification of APOE‐positive area within IBA1‐positive microglia surrounding plaques (APOE/IBA1 area) (*n* = 9–12); (J, K) Lilrb4a mRNA expression was analyzed by qPCR in cortical tissues from C57 and 5xFAD mice of different ages (*n* = 5–8); Unless otherwise specified, experiments not shown as sex‐separated in the figures were performed using male mice only (Applicable to all figure legends). Data are presented as mean ± standard error of the mean (SEM). Each dot represents one mouse. Statistical significance was determined by unpaired two‐tailed Student's *t* test (F), one‐way ANOVA (J,K), or two‐way ANOVA (I), as appropriate. ^*^ *p* <0.05, ^**^ *p* <0.01.

As research on the relationship between LILRB4 and APOE deepens, accumulating evidence suggests a potential interaction between these two proteins [20, 21]. However, some studies have indicated that LILRB3, a member of the same family, may instead be the receptor for APOE4 [22]. To address this, we first performed co‐immunoprecipitation (Co‐IP) assays in HEK293T cells co‐transfected with human APOE4 and either Flag‐tagged human LILRB4 or Flag‐tagged human LILRB3. Anti‐Flag pulldown followed by immunoblotting for human APOE showed that APOE4 showed stronger Co‐IP with LILRB4 than with LILRB3 under these overexpression conditions (Figure 1A,B). We next examined this interaction under endogenous conditions by reciprocal Co‐IP in microglia isolated from *APOE3* and *APOE4* mice 24 h after intraperitoneal lipopolysaccharide (LPS) injection (3 mg/kg) (Figure S1A). Consistent with the overexpression system, endogenous APOE4 displayed a stronger association with Lilrb4a than did APOE3 (Figure 1C,D). In parallel, cortical Lilrb4a immunoreactivity was higher in LPS‐treated *APOE4* mice than in *APOE3* mice (Figure 1E,F). These Co‐IP data support protein association in the tested contexts but do not by themselves establish direct binding affinity or define APOE4–Lilrb4a as an initiating receptor–ligand event.

Subsequently, to investigate the effects of *Lilrb4a* deletion in an AD mouse model, we knocked out the *Lilrb4a* gene in 5EL transgenic mice, generating the 5ELKO mice (see Experimental section). The experimental design is illustrated (Figure 1G). The successful knockout of *Lilrb4a* in the constructed mice was confirmed by qPCR, WB, and IHC (Figure S1B–D). We then performed confocal analysis of X‐34/APOE/IBA1 staining in 5EL and 5ELKO mice and found that *Lilrb4a* deletion significantly reduced APOE immunoreactivity within plaque‐associated microglia (Figure 1H,I), further supporting the relevance of APOE–Lilrb4a association in plaque‐associated microglia under this experimental context. Notably, *Lilrb4a* mRNA also increased with age in the brains of C57 and 5xFAD mice, with a more pronounced elevation in 5xFAD mice (Figure 1J,K), consistent with previous reports [13, 14, 15].

### 2.2 *Lilrb4a* Deletion Reduces Amyloid Plaque Burden and Alleviates CAA in Human *APOE4* Alzheimer's Mice

To determine whether *Lilrb4a* deletion affects baseline behavior, we generated *APOE4* knock‐in mice with or without *Lilrb4a* deletion (EL and ELKO). We conducted behavioral tests on 4.5‐month‐old EL and ELKO mice (*n* = 10, five males and five females per group). These tests included the Y‐maze (Figure S1E,F), elevated plus maze (EPM) (Figure S1G,H), and open field test (OFT) (Figure S1I,J). No significant behavioral differences were detected between ELKO and EL mice. This suggests that the absence of *Lilrb4a* did not affect memory, anxiety, or other behaviors in normal mice.

**FIGURE 2:**
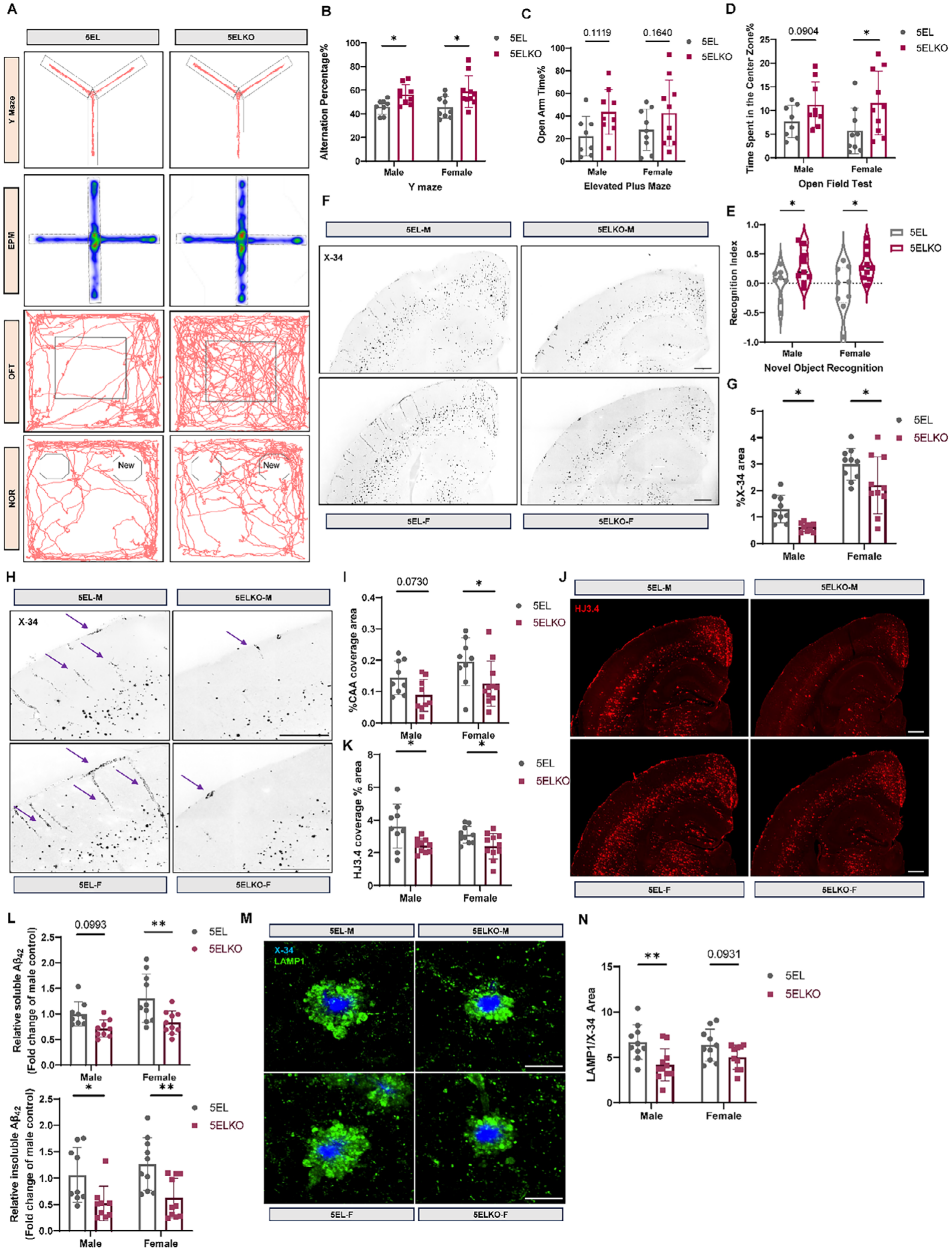
*Lilrb4a* deletion ameliorates behavioral deficits, CAA, and amyloid burden‐related pathology in 5EL mice. (A–E) Representative movement traces from the Y‐maze, EPM, OFT, and NOR task in 5EL and 5ELKO mice (A), quantification of spontaneous alternation in the Y‐maze (B), time spent in the open arms in the EPM (C), time spent in the center zone in the OFT (D), and recognition index in the NOR task (E) in 5EL and 5ELKO mice (*n* = 10, 5 males and 5 females); (F) Representative X‐34 histochemistry of cortical sections from male and female 5EL and 5ELKO mice (10×, scale bar = 500 µm); (G) Quantification of X‐34 positive plaque area in the cortex (*n* = 9–12); (H) Representative X‐34 histochemistry of cortical sections showing CAA like vascular amyloid deposits, indicated by arrows (10×, scale bar = 200 µm); (I) Quantification of X‐34‐positive CAA coverage area in the cortex (*n* = 9–12); (J) Representative HJ3.4 immunofluorescence staining of cortical sections from 5EL and 5ELKO mice (10×, scale bar = 500 µm); (K) Quantification of HJ3.4 positive area in the cortex (*n* = 9–12); (L) ELISA quantification of Aβ_42_ in PBS‐soluble (top) and guanidine‐soluble (bottom) cortical fractions from 5EL and 5ELKO mice (*n* = 9–10); (M) Representative high magnification confocal images of X‐34 (blue) and LAMP1 (green) co‐staining in cortical sections from 5EL and 5ELKO mice, showing dystrophic neurites surrounding plaques (60× oil, scale bar = 20 µm); (N) Quantification of dystrophic neurite area around plaques, expressed as the LAMP1/X‐34 area ratio (*n* = 9–12). Data are presented as mean ± SEM. Statistical significance was determined by two‐way ANOVA followed by post hoc multiple‐comparison tests. ^*^ *p* <0.05, ^**^ *p* <0.01.

We next asked whether *Lilrb4a* deletion modifies behavioral deficits in 5EL mice. Relative to 5EL mice, 5ELKO mice exhibited increased spontaneous alteration in the Y‐maze, consistent with improved working memory (Figure 2A,B). Although time spent in the open arms of the EPM was unchanged (Figure 2A,C), 5ELKO mice spent more time in the center zone of the OFT, suggesting reduced anxiety‐like behavior (Figure 2A,D). 5ELKO mice also showed a higher recognition index in the novel object recognition (NOR) task, indicating improved recognition memory (Figure 2A,E). Together, these findings indicate that loss of *Lilrb4a* is associated with improved performance in selected behavioral readouts in the *APOE4*‐driven 5EL model.

Amyloid plaque deposition is a core pathological feature of AD. We selected brain sections from each mouse at the same spatial level and stained them with X‐34 to label fibrillar Aβ (Figure 2F). Mice were analyzed separately by sex, and the X‐34–positive area in the cortex was expressed as a percentage of total cortical area (Figure 2G). In both males and females, 5ELKO mice showed significantly lower plaque burden than 5EL littermates, indicating that *Lilrb4a* deletion reduces amyloid plaque accumulation. Consistent with this, CAA burden was also significantly reduced in 5ELKO mice (Figure 2H,I).

We purified antibodies from the HJ3.4 hybridoma cell line, provided by David Holtzman's lab at Washington University School of Medicine, and used the HJ3.4 antibody to detect Aβ (Figure 2J). We found that the area of Aβ coverage in the brains of 5ELKO mice was reduced compared to that of 5EL mice (Figure 2K). Moreover, we observed a decrease in the soluble and insoluble Aβ_42_ fractions within the cortex, which corresponds with the decrease in amyloid plaques (Figure 2L). In AD patients, the deposition of amyloid plaques in the brain leads to the formation of dystrophic neurites (DNs) around the plaques, which represent damage to the surrounding neurons [23]. Next, we performed co‐staining of lysosome‐associated membrane protein 1 (LAMP1) and X‐34 to visualize the coverage of DNs around the plaques (Figure 2M). Our results showed that the area of DNs surrounding plaques was significantly lower in male 5ELKO mice compared to 5EL mice, and there was also a notable decreasing trend in female mice (Figure 2N). These findings indicate that knocking out *Lilrb4a* effectively attenuates neuronal damage.

In previous results, we observed a significant reduction in Aβ, plaque deposition, and DNs around the plaques. To explore whether this reduction was due to decreased APP cleavage, we performed WB analysis on brain tissue using the A8717 antibody to detect APP‐C ‐terminal cleavage products (Figure S2A). Our results showed no changes in APP full‐length (APP‐FL), β‐CTF, or α‐CTF between the two groups of mice (Figure S2B). We also used the 6E10 antibody to detect oligomeric Aβ (o‐Aβ) in the brain (Figure S2C) and found no significant difference between the two groups (Figure S2D). These results indicate that knocking out *Lilrb4a* does not affect APP cleavage. Together with the unchanged APP processing measures, these findings are more consistent with altered amyloid handling/clearance than with altered Aβ production.

### 2.3 *Lilrb4a* Knockout Enhances Microglial Phagocytosis, Amoeboid Transformation, and Astrocytic Engagement With Plaques and CAA

**FIGURE 3:**
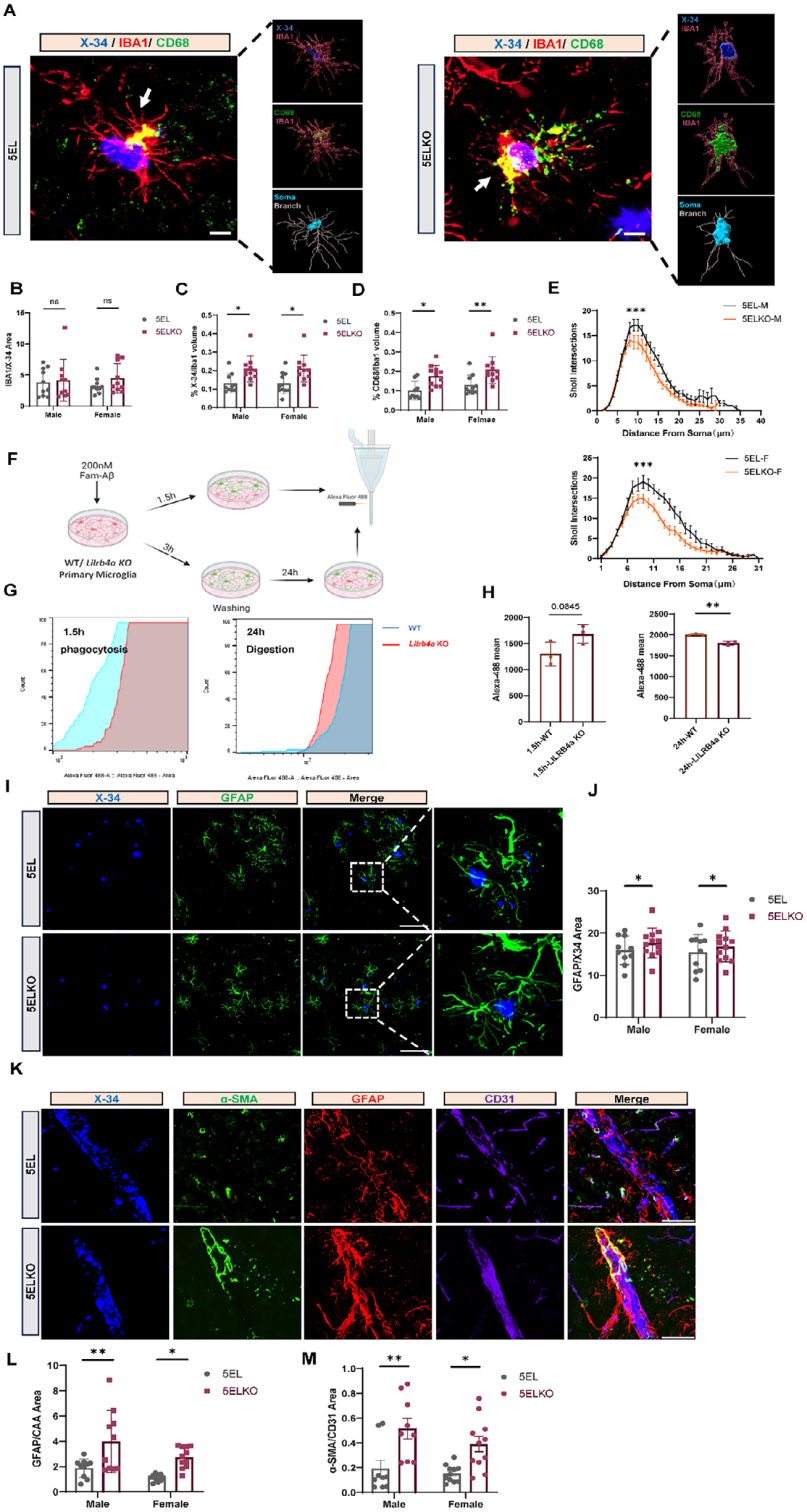
*Lilrb4a* knockout enhances microglial phagocytosis, amoeboid transformation, and astrocytic engagement with plaques and CAA. (A) Representative confocal images of X‐34 (blue), IBA1 (red), and CD68 (green) staining in cortical sections from 5EL and 5ELKO mice; right panels show Imaris‐based 3D reconstructions of X‐34/IBA1, CD68/IBA1, and microglial soma/branch morphology (60× oil, scale bar = 5 µm); (B) Quantification of plaque‐associated microglial coverage, expressed as the IBA1/X‐34 area ratio (*n* = 10–12); (C) Quantification of amyloid signal within microglia, expressed as X‐34/IBA1 volume (%) (*n* = 10–12); (D) Quantification of lysosomal activity content within microglia, expressed as CD68/IBA1 volume (%) (*n* = 10–12); (E) Sholl analysis of plaque‐associated microglia in male and female 5EL and 5ELKO mice (*n* = 10–12); (F) Schematic diagram of the phagocytosis and degradation assays in primary microglia from wild‐type (WT) and *Lilrb4a* KO mice using FAM‐labeled (Alexa‐488) Aβ and flow cytometry; (G) Representative flow cytometry histograms showing intracellular Alexa‐488 fluorescence intensity after 1.5 h phagocytosis and 24 h degradation in WT and *Lilrb4a* KO primary microglia; (H) Quantification of mean Alexa‐488 fluorescence intensity after 1.5 h phagocytosis (left) and 24 h degradation (right) (*n* = 3); (I) Representative confocal images of X‐34 (blue) and GFAP (green) staining in cortical sections from 5EL and 5ELKO mice, showing astrocytic engagement around plaques (40×, scale bar = 5 µm); (J) Quantification of astrocyte coverage around plaques, expressed as the GFAP/X‐34 area ratio (*n* = 10–12); (K) Representative confocal images of X‐34 (blue), α‐SMA (green), GFAP (red), and CD31 (purple) staining in CAA lesions from 5EL and 5ELKO mice (40×, scale bar = 10 µm); (L) Quantification of astrocyte coverage around CAA, expressed as the GFAP/CAA area ratio (*n* = 10–12); (M) Quantification of vascular smooth muscle coverage in CAA lesions, expressed as the α‐SMA/CD31 area ratio (*n* = 10–12). Data are presented as mean ± SEM. Statistical significance was determined by two‐way ANOVA (B,C,D,,L,M) or unpaired two‐tailed Student's t test (E,H), as appropriate. ns, not significant, ^*^ *p* <0.05, ^**^ *p* <0.01, ^***^ *p* <0.001.

Microglia, the resident immune cells of the brain, are key mediators of phagocytic clearance of diverse substrates—including Aβ plaques—and are central to AD pathogenesis [24]. In AD, microglia become activated, transitioning from a static, ramified state to an “Amoeboid” morphology, which enhances their phagocytic capacity [25, 26, 27, 28]. To investigate the impact of *Lilrb4a* knockout on microglial morphology and function, we reconstructed microglia surrounding plaques and analyzed the lysosomal marker cluster of differentiation 68 (CD68), which reflects phagocytic activity (Figure 3A). Our results showed that the number of microglia surrounding plaques remained unchanged (Figure 3B). Consistent with our hypothesis, microglia in 5ELKO mice contained a higher proportion of X‐34 within their lysosomes (Figure 3C), suggesting that *Lilrb4a* knockout enhances microglial phagocytosis of plaques. Furthermore, CD68 levels were significantly elevated in 5ELKO microglia compared to 5EL microglia (Figure 3D), indicating increased lysosomal activity. Additionally, we performed detailed morphological analyses of microglia. Our results revealed a substantial reduction in branch complexity, total branch length, and the number of branch terminal points in 5ELKO mice compared to 5EL mice (Figure 3E and Figure S3A–E). These findings suggest that knocking out *Lilrb4a* leads to a reduction in microglial complexity, making them more “Amoeboid‐like,” while simultaneously enhancing their plaque phagocytic capacity in the AD mouse model.

To determine whether the microglial changes observed in vivo are consistent in vitro, we isolated primary microglia from P2 C57 and L4KO mice and assessed their phagocytic and degradative capacity using FAM‐Aβ and flow cytometry (Figure 3F). Our results demonstrated that *Lilrb4a* knockout significantly enhanced both the phagocytosis and digestion of FAM‐Aβ in primary microglia (Figure 3G,H and Figure S3F,G), consistent with our in vivo findings. In addition, although microglia from 5ELKO mice exhibited enhanced engulfment of amyloid plaques, their uptake of postsynaptic density protein 95 (PSD95) was significantly reduced (Figure S3H,I), accompanied by increased PSD95 protein levels in brain tissue (Figure S3J,K). This pattern is consistent with the reduced neuritic injury and improved behavioral performance observed in 5ELKO mice (Figure 2A–E,M,N).

Astrocytes account for approximately 20%–40% of brain cells, displaying substantial heterogeneity in structure and function [29, 30, 31]. Given their established activation in AD around amyloid deposits [32, 33, 34], we quantified astrocyte coverage in parenchymal plaque and vascular amyloid (CAA) regions in 5EL and 5ELKO mice. Astrocyte coverage surrounding parenchymal plaques was significantly increased in 5ELKO mice compared to 5EL controls (Figure 3I,J). Similarly, astrocyte coverage at CAA sites was markedly elevated following *Lilrb4a* knockout (Figure 3K,L). Concomitantly, vascular smooth muscle coverage in CAA lesions was also increased (Figure 3M). In contrast, microglia did not show increased association with CAA (Figure S3L,M). These observations indicate differential glial responses at parenchymal versus vascular amyloid sites in this model.

### 2.4 Pharmacological Activation of PPAR‑γ Recapitulates Microglial Clearance Enhancement

**FIGURE 4:**
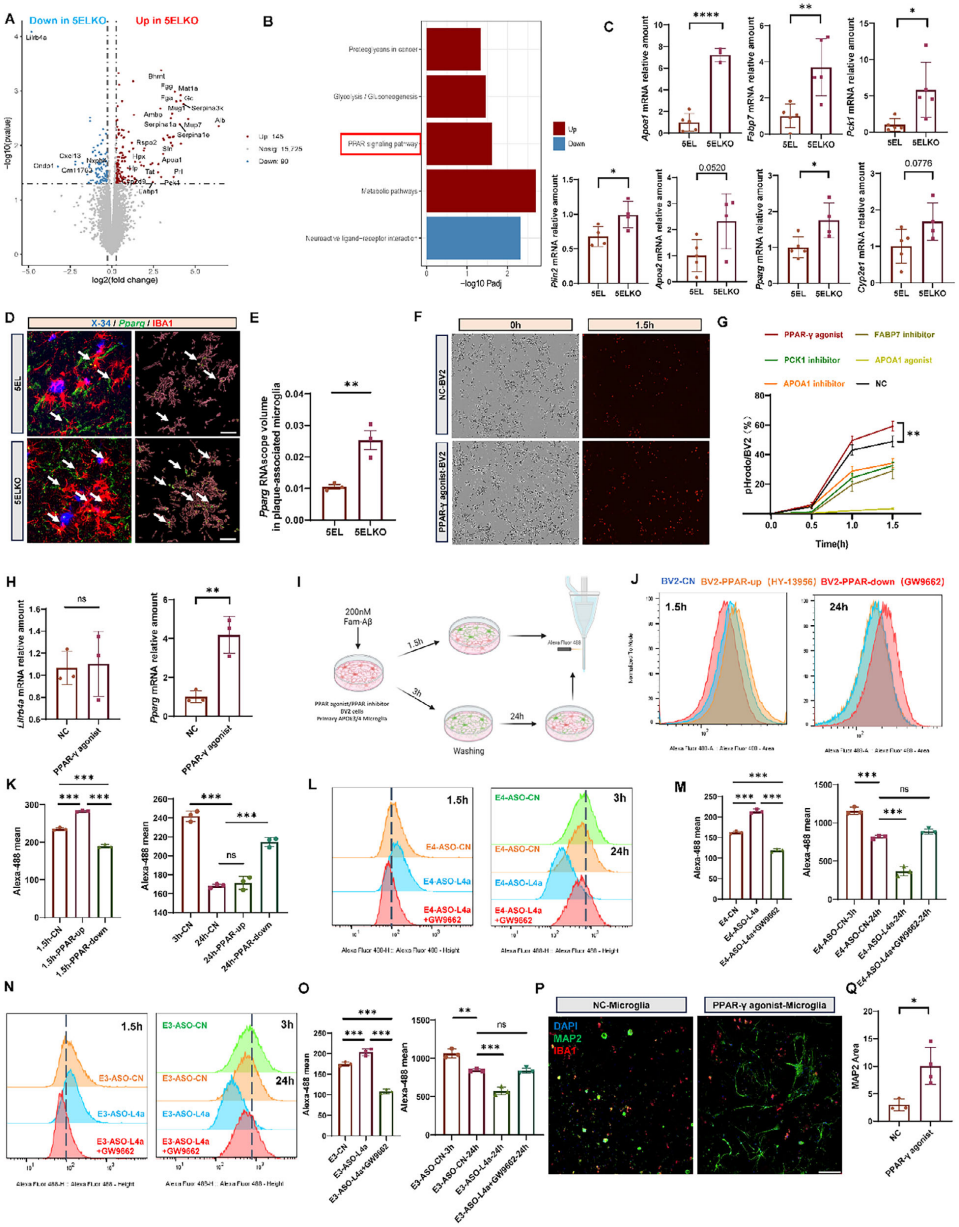
Exploratory bulk RNA‐seq nominates a PPAR‐related signature, and PPAR‑γ activation phenocopies clearance‐associated outcomes. (A) Volcano plot of bulk RNA‐seq data from thalamic tissue of 5EL and 5ELKO mice; colored points indicate genes meeting an exploratory threshold of nominal *p* <0.05 and absolute fold change >1.2; (B) KEGG pathway enrichment analysis of transcriptomic alterations in 5ELKO versus 5EL mice; (C) qPCR validation of selected genes related to the PPAR signaling pathway, including *Apoa1*, *Fabp7*, *Pck1*, *Plin2*, *Apoa2*, *Pparg*, *Cyp2e1* in thalamus from 5EL and 5ELKO mice (*n* = 4–5); (D) Representative RNAscope images of *Pparg* mRNA in brain sections from 5EL and 5ELKO mice, combined with X‐34 and IBA1 co‐staining (left) and Imaris‐based reconstruction (right). White arrows indicate PPAR‐γ‐positive signals within microglia (60× oil, scale bar = 20 µm); (E) Quantification of *Pparg* RNAscope signal volume within plaque‐associated microglia in 5EL and 5ELKO mice; (F) Representative Incucyte images of pHrodo‐labeled Aβ uptake in *BV2* cells treated with vehicle or a PPAR‐γ agonist at 0 h and 1.5 h; (G) Quantification of the phagocytosis rate in *BV2* cells under the indicated agonist or inhibitor treatments, expressed as the percentage of pHrodo‐positive area relative to total cell area (*n* = 3); (H) qPCR analysis of *Lilrb4a* and *Pparg* expression in *BV2* cells treated with vehicle or a PPAR‐γ agonist (*n* = 3); (I) Schematic diagram of the FAM‐labeled Aβ phagocytosis and degradation assays in BV2 cells; (J) Representative flow cytometry histograms showing intracellular Alexa‐488 fluorescence in *BV2* cells after 1.5 h phagocytosis and after 3 h loading followed by 24 h degradation under the indicated PPAR‐γ agonist or inhibitor conditions; (K) Quantification of mean intracellular Alexa‐488 fluorescence in *BV2* cells after 1.5 h phagocytosis (left) and after 24 h degradation (right) (*n* = 3); (L) Representative flow cytometry histograms showing intracellular Alexa‐488 fluorescence in sorted primary microglia from LPS‐treated *APOE4* mice under the indicated ASO and PPAR‐γ inhibitor conditions. Left, phagocytosis assay, in which cells were incubated with FAM‐Aβ and analyzed after 1.5 h to assess uptake. Right, degradation assay, in which cells were first allowed to internalize FAM‐Aβ for 3 h, then switched to substrate‐free medium, and analyzed 24 h later to assess residual intracellular signal; (M) Quantification of mean intracellular Alexa‐488 fluorescence in *APOE4* microglia in the phagocytosis (left) and degradation (right) assays (*n* = 3); (N,O) Corresponding representative histograms and quantification for sorted primary microglia from LPS‐treated *APOE3* mice under identical assay conditions and treatment paradigms, with phagocytosis measured at 1.5 h and degradation measured 24 h after 3 h loading with FAM‐Aβ (*n* = 3); (P) Representative immunofluorescence images of WT primary neuron–microglia co‐cultures treated with vehicle‐ or PPAR‐γ agonist‐treated microglia, showing DAPI (blue), MAP2 (green), and IBA1 (red) (10×, scale bar = 200 µm); (Q) Quantification of neuronal survival in co‐culture, measured as MAP2‐positive area (*n* = 8–11). Data are presented as mean ± SEM. Statistical significance was determined by unpaired two‐tailed Student's *t* test (C, E, H, and Q) or one‐way ANOVA (G, K, M, and O), as appropriate. ns, not significant. ^*^ *p* <0.05, ^**^ *p* <0.01, ^***^ *p* <0.001.

To explore the potential mechanisms by which *Lilrb4a* influences the AD phenotype, we performed bulk RNA sequencing on brain thalamic tissue from 5EL and 5ELKO mice. Based on X‐34 quantification in the thalamus (Figure S4A), this region was selected for bulk RNA‐seq analysis. Given the cellular heterogeneity of bulk brain tissue, these data were used primarily to nominate pathway‐level candidates for targeted follow‐up rather than as stand‐alone evidence of microglia‐specific transcriptional regulation in vivo. We performed principal component analysis on the two groups (Figure S4B). Using an exploratory gene‐level threshold of nominal *p* <0.05 and absolute fold change >1.2, we observed 145 upregulated and 90 downregulated genes in 5ELKO tissue relative to 5EL tissue (Figure 4A). KEGG pathway enrichment analysis revealed enrichment of the PPAR signaling pathway, Glycolysis/Gluconeogenesis, and broader metabolic pathways in 5ELKO mice (Figure 4B), which guided our subsequent mechanistic validation in primary microglia and microglial cell lines.

Here, we focused on the changes in the PPAR signaling pathway. We selected several genes that either regulate or are regulated by the PPAR signaling pathway: *Apolipoprotein A1 (APOA1)*, *Fatty Acid Binding Protein 7 (Fabp7)*, *Phosphoenolpyruvate Carboxykinase 1 (PCK1)*, an important regulatory enzyme in gluconeogenesis, *Perilipin 2 (Plin2), Apolipoprotein A2 (Apoa2)*, *Pparg*, and *Cytochrome P450 family 2 subfamily E member 1 (Cyp2e1)*. We validated the expression of these genes using qPCR (Figure 4C). Because *PPAR‐γ* is a central PPAR‐family transcription factor in microglia, we next anchored this pathway directly to microglia in vivo. RNAscope localized *Pparg* signal to plaque‐associated IBA1‐positive microglia (Figure 4D,E). In parallel, sorted microglia isolated from 5EL and 5ELKO mice exhibited corresponding alterations in *Pparg* and key PPAR‐associated genes, including *Cyp2e1*, *Lipoprotein Lipase (Lpl)*, *ATP‐binding cassette transporter A1 (Abca1)*, and *Cluster of differentiation 36 (Cd36)* (Figure S4C).

Together, these data were consistent with increased engagement of PPAR‐related pathways in *Lilrb4a*‐deficient tissue. To test whether PPAR‐γ activation alone could recapitulate aspects of *Lilrb4a* suppression, *BV2* microglial cells were treated in vitro for 24 h with a PPAR‐γ agonist, APOA1 agonist, FABP7 inhibitor, PCK1 inhibitor, APOA1 inhibitor, or DMSO as a control, followed by a 6 h LPS challenge. pHrodo‐labeled Aβ was then added to assess phagocytic capacity (Figure 4F). Among these treatments, *BV2* cells treated with the PPAR‐γ agonist showed a significant increase in phagocytic capacity (Figure 4G), while *Lilrb4a* expression remained unchanged (Figure 4H). Furthermore, flow cytometry analysis revealed that the addition of a PPAR‐γ agonist also enhanced *BV2* cell phagocytosis and degradation of FAM‐Aβ (Figure 4I–K), consistent with the results observed in L4KO primary microglia (Figure 3F–H). In addition, to determine whether the microglial changes mediated by *Lilrb4a* deficiency were dependent on *APOE* isoform, we isolated microglia from LPS‐treated *APOE3* and *APOE4* mice and treated them with either ASO‐L4a or control ASO, followed by PPAR‐γ inhibitor treatment in vitro. Under the same experimental conditions, FAM‐Aβ was then added, and microglial phagocytic and degradative capacities were assessed by flow cytometry. In both *APOE3* and *APOE4‐*derived microglia, *Lilrb4a* knockdown enhanced Aβ phagocytosis and degradation, whereas PPAR‐γ inhibition abolished the enhancement of Aβ phagocytosis and degradation induced by *Lilrb4a* knockdown (Figure 4L–O). The efficacy of ASO‐L4a and the associated treatment paradigm was independently validated (Figure S4C,D).

**FIGURE 5:**
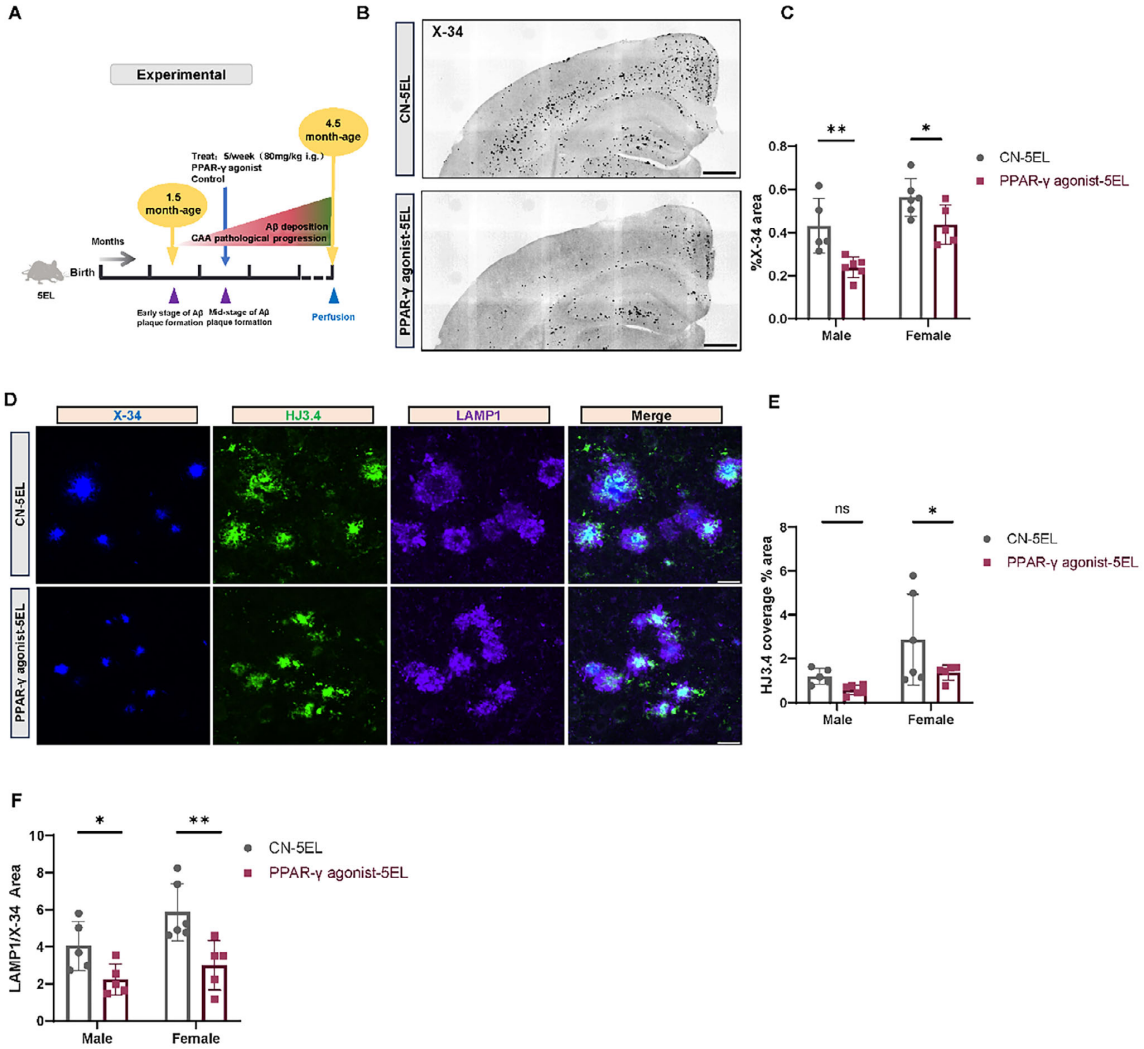
PPAR‐γ agonist treatment attenuates amyloid‐related pathology in 5EL mice. (A) Schematic diagram of the experimental design; (B) Representative X‐34 histochemistry of cortical sections from untreated control (CN‐5EL) and PPAR‐γ agonist‐treated (PPAR‐γ agonist‐5EL) mice (10×, scale bar = 500 µm); (C) Quantification of X‐34‐positive plaque area in the cortex (*n* = 5–6); (D) Representative high‐magnification confocal images of X‐34 (blue), HJ3.4 (green), and LAMP1 (red) staining in cortical sections from CN‐5EL and PPAR‐γ agonist‐5EL mice (60× oil, scale bar = 20 µm); (E) Quantification of HJ3.4‐positive area in the cortex (*n* = 5–6); (F) Quantification of dystrophic neurite area around plaques, expressed as the LAMP1/X‐34 area ratio (*n* = 5–6). Data are presented as mean ± SEM. Statistical significance was determined by two‐way ANOVA. ^*^ *p* <0.05, ^**^ *p* <0.01.

Next, we co‐cultured primary WT microglia from P2 mice with WT neurons from E17 mice. Cells were treated with either a PPAR‐γ agonist or DMSO for 24 h, followed by LPS treatment for 6h to induce neuroinflammation, and then subjected to DAPI/MAP2/IBA1 staining (Figure 4P). The results demonstrated that neuronal survival was significantly enhanced in cells treated with the PPAR‐γ agonist (Figure 4Q). Concurrently, in vivo studies, we treated 5EL mice with a PPAR‐γ agonist (Figure 5A). At 4.5 months of age, PPAR‐γ agonist‐treated mice exhibited reduced Aβ‐related pathology—including plaque burden (Figure 5B,C), Aβ coverage (Figure 5D,E), and DNs (Figure 5D,F) compared with vehicle controls. These findings indicate that pharmacological PPAR‐γ activation can recapitulate key clearance‐associated phenotypes in vitro and reduce amyloid‐related pathology in vivo. We interpret the in vivo pioglitazone experiment primarily as a pharmacological pathway perturbation rather than as evidence of microglia‐specific target engagement.

### 2.5 SHP2/NF‐κB and JAKSTAT Pathways are Modulated to Favor PPAR‐Driven Anti‐Inflammatory Programs

**FIGURE 6:**
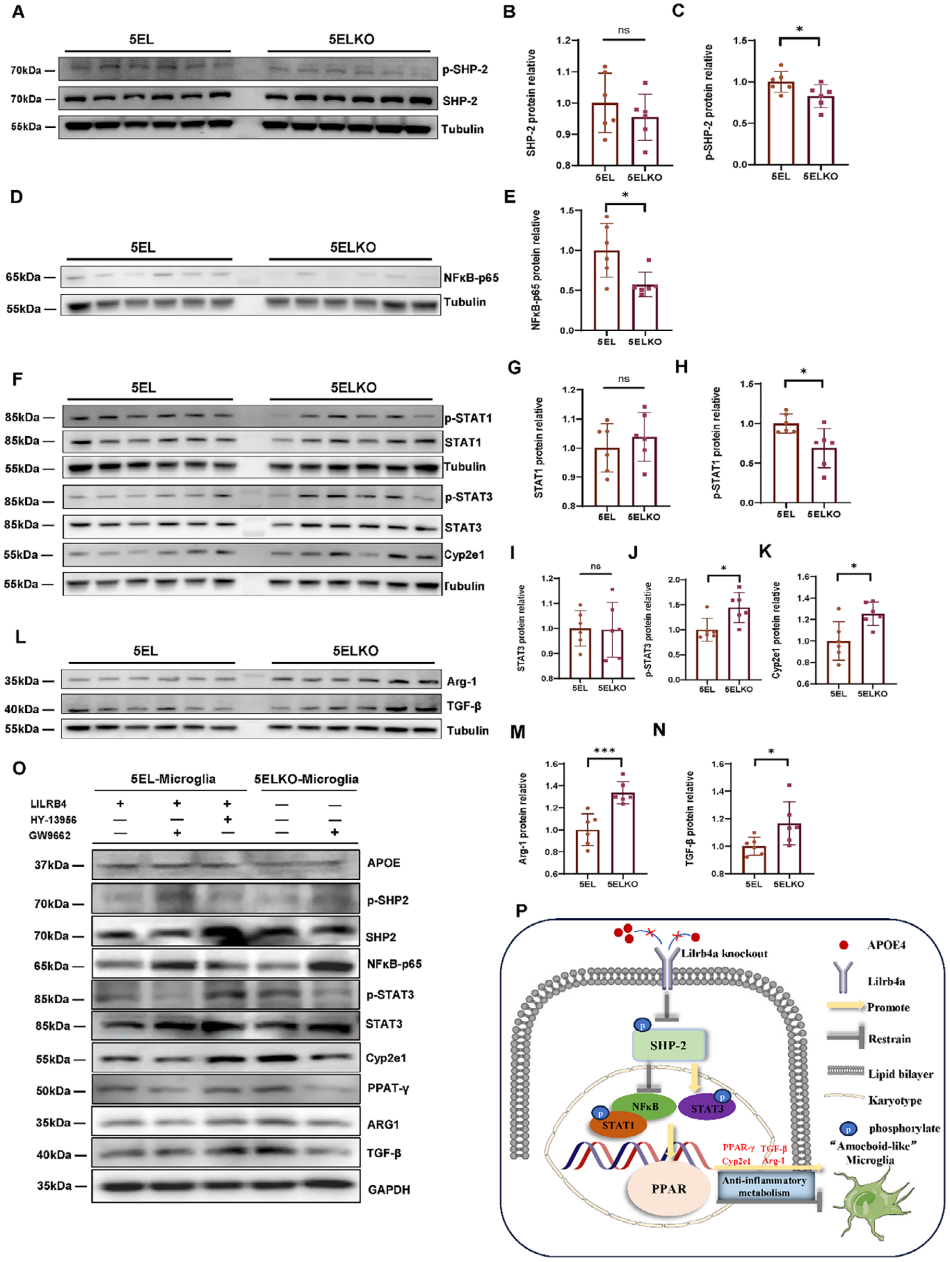
Signaling changes associated with *Lilrb4a* suppression that are consistent with enhanced PPAR‐linked anti‐inflammatory programs. (A) Representative immunoblots of p‐SHP2 and SHP2 in prefrontal cortical lysates from 5EL and 5ELKO mice; (B,C) Quantification of p‐SHP2 (B) and SHP2 (C) protein levels (*n* = 6); (D) Representative immunoblot of NF‐κB p65 in prefrontal cortical lysates from 5EL and 5ELKO mice; (E) Quantification of NF‐κB p65 protein levels (*n* = 6); (F) Representative immunoblots of STAT1, p‐STAT1, STAT3, p‐STAT3, and CYP2E1 in prefrontal cortical lysates from 5EL and 5ELKO mice; (G–K) Quantification of STAT1 (G), p‐STAT1 (H), STAT3 (I), p‐STAT3 (J), and Cyp2e1 (K) protein levels (*n* = 6); (L) Representative immunoblots of ARG1 and TGF‐β in prefrontal cortical lysates from 5EL and 5ELKO mice; (M, N) Quantification of ARG1 (M) and TGF‐β (N) protein levels (*n* = 6); (O) Analysis of sorted 5EL and 5ELKO primary microglia treated with the PPAR‐γ agonist (HY‐13956) or the PPAR‐γ inhibitor (GW9662); (P) Working model summarizing the proposed signaling changes associated with *Lilrb4a* suppression in microglia. Data are presented as mean ± SEM. Statistical significance was determined by an unpaired two‐tailed Student's *t*‐test. ns, not significant, ^*^ *p* <0.05, ^***^ *p* <0.001.

To elucidate the downstream mechanisms by which *Lilrb4a* regulates microglial states, we examined the phosphorylation levels of SHP‐2, a mediator of LILRB4 signaling [19]. We found that p‐SHP‐2 levels were significantly reduced in 5ELKO mice (Figure 6A–C). Previous studies have shown that the NF‐κB pathway is positively regulated by p‐SHP‐2 [23, 35]. Correspondingly, NF‐κBp65 levels were significantly downregulated in 5ELKO mice (Figure 6D,E). Moreover, the JAKSTAT axis also exhibited selective modulation: phosphorylation of STAT1 was decreased, whereas phosphorylation of STAT3 was increased (Figure 6F–J). As both NF‐κB and pro‐inflammatory STAT1 activity are established repressors of PPAR‑γ–mediated transcription, we assessed protein markers of PPAR pathway activity [36, 37, 38]. *Lilrb4a* deletion increased expression of the PPAR‐positively regulated enzyme Cyp2e1 (Figure 6K) [39], along with the canonical anti‐inflammatory effectors arginase‐1 (Arg‐1) and transforming growth factor beta (TGF‐β (Figure 6L–N). In sorted primary microglia, pharmacological modulation further supported this relationship between Lilrb4a status and PPAR pathway engagement (Figure 6O). Collectively, these data support a model in which Lilrb4a suppression alters SHP‐2‐, NF‐κB‐, and JAK–STAT‐related signaling in a manner consistent with enhanced PPAR‐associated anti‐inflammatory and clearance programs (Figure 6P).

### 2.6 ASO‐Mediated *Lilrb4a* Suppression Recapitulates Genetic Knockout Effects

**FIGURE 7:**
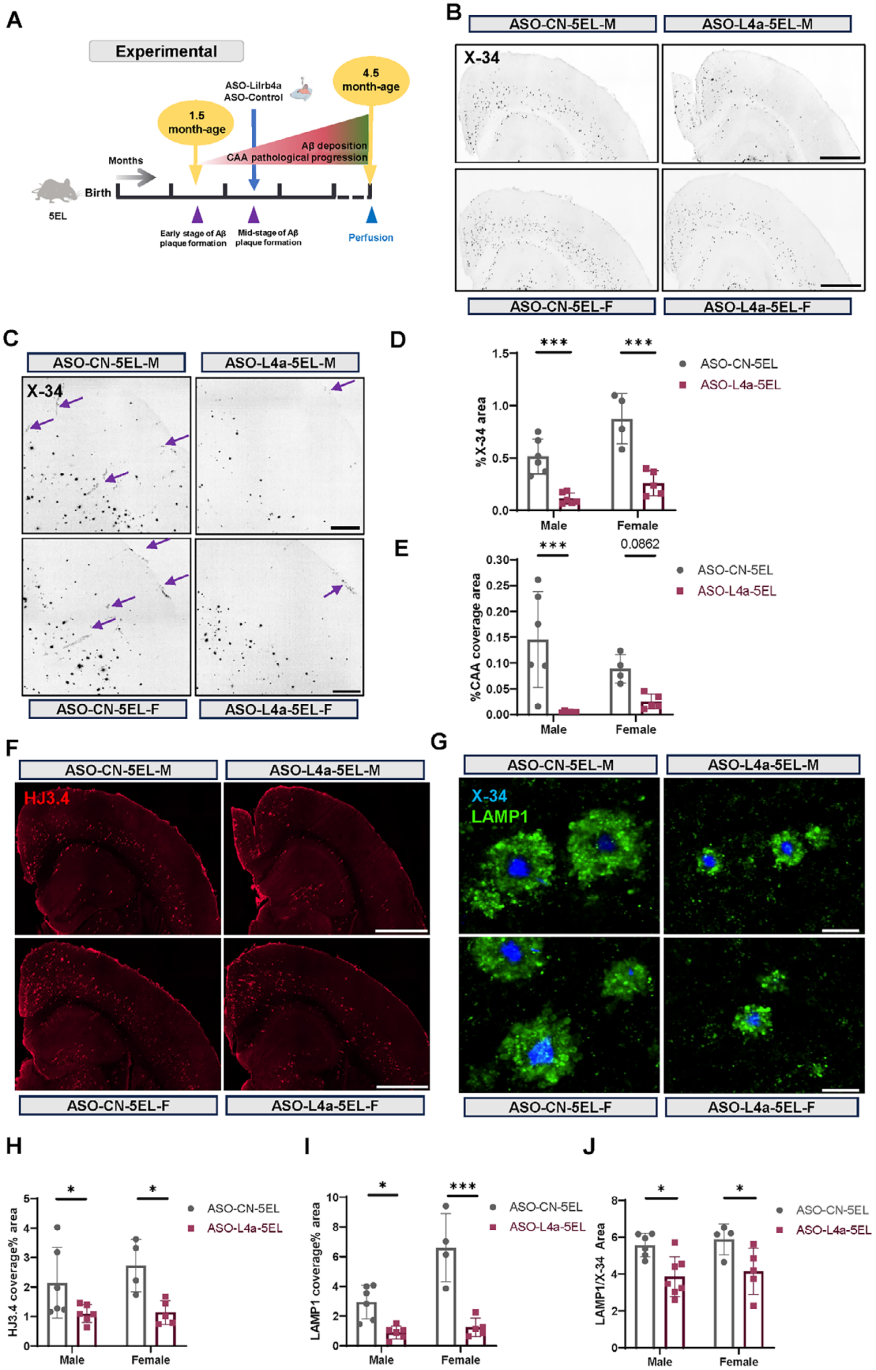
ASO‐mediated *Lilrb4a* knockdown attenuates CAA and amyloid burden‐related pathology in 5EL mice. (A) Schematic diagram of the experimental design; (B) Representative X‐34 histochemistry of cortical sections from ASO‐CN‐5EL and ASO‐L4a‐5EL mice showing plaque burden (10×, scale bar = 500 µm); (C) Representative X‐34 histochemistry of cortical sections from ASO‐CN‐5EL and ASO‐L4a‐5EL mice showing CAA‐related vascular amyloid deposits (Purple arrow) (10×, scale bar = 200 µm); (D,E) Quantification of X‐34‐positive plaque area (D) and X‐34‐positive CAA coverage area (E) in the cortex (*n* = 4–7); (F) Representative HJ3.4 immunofluorescence staining of cortical sections from ASO‐CN‐5EL and ASO‐L4a‐5EL mice (10×, scale bar = 500 µm); (G) Representative high‐magnification confocal images of X‐34 (blue) and LAMP1 (green) staining in cortical sections from ASO‐CN‐5EL and ASO‐L4a‐5EL mice, showing dystrophic neurites surrounding plaques (60× oil, scale bar = 20 µm); (H, I) Quantification of HJ3.4‐positive area (H) and LAMP1‐positive area in the cortex (I) (*n* = 4–7); (J) Quantification of LAMP1/X‐34 area from three randomly selected locations within the cortical region above the hippocampal subiculum per brain section, averaged to generate one value per animal (*n* = 4–7). Data are presented as mean ± SEM. Statistical significance was determined by two‐way ANOVA. ^*^ *p* <0.05, ^**^ *p* <0.01.

Finally, to test whether ASO‐mediated *Lilrb4a* suppression could phenocopy the genetic findings in vivo, we examined the effects of ASO‐mediated *Lilrb4a* knockdown in 5EL mice. We administered ASO designed against the *Lilrb4a* sequence to 2.5‐month‐old 5EL mice. Brain tissue samples were collected at 4.5 months of age for pathological analysis and validation of ASO efficacy (Figure 7A and Figure S5A–C). As expected, our results demonstrated a significant reduction in plaque burden following ASO treatment, regardless of sex (Figure 7B,D). Similarly, CAA levels were also reduced (Figure 7C,E). Furthermore, the overall amount of Aβ plaque deposition was decreased (Figure 7F,H), and there was a notable reduction in DNs (Figure 7G,I,J).

Together, these findings indicate that ASO‐mediated *Lilrb4a* suppression is sufficient to reproduce key aspects of the genetic knockout phenotype in 5EL mice. These data show that in vivo ASO‐mediated *Lilrb4a* perturbation can phenocopy selected aspects of the genetic knockout phenotype in this model.

## 3 Conclusion

This work identifies *Lilrb4a* as an *APOE4*‐associated microglial checkpoint candidate and supports a role for Lilrb4a suppression in amyloid clearance–related phenotypes in vivo. In a human *APOE4* knock‐in 5xFAD background (5EL), genetic ablation or ASO‐mediated suppression of *Lilrb4a* markedly reduced plaque burden and neuritic dystrophy and mitigated CAA, while pharmacological activation of PPAR‐γ further amplified microglial phagocytosis and protected neurons under inflammatory stress (Figure 2F–N, Figure 5B–F, Figure 7B–J).

Mechanistically, the data support a model linking Lilrb4a to SHP‐2, NF‐κB, JAK‐STAT, and PPAR‐associated signaling changes relevant to microglial activation and metabolism. In Co‐IP assays, LILRB4 showed stronger association with APOE4 than LILRB3 in the heterologous overexpression system, and endogenous APOE4 in isolated microglia also displayed stronger association with LILRB4 than APOE3 (Figure 1A–D). Importantly, these results support protein association rather than proving direct binding affinity, and future orthogonal approaches will be needed to define the molecular interface more rigorously. We therefore interpret Lilrb4a in this study as an APOE4‐associated inhibitory checkpoint candidate. Loss of *Lilrb4a* decreased p‐SHP2 and NF‐κB‐p65, shifted the STAT balance (p‐STAT1 down, p‐STAT3 up), and increased anti‐inflammatory effectors (Arg1, TGF‐β) and metabolic enzymes (Cyp2e1) (Figure 6A–N). In parallel, bulk RNA‐seq served as exploratory discovery support and nominated PPAR‐related and broader metabolic pathways for targeted follow‐up. These pathway‐level leads were then anchored in vivo by *Pparg* RNAscope in plaque‐associated microglia and by qPCR validation of selected PPAR‐related genes in sorted microglia (Figure 4A–E and Figure S4C). Functionally, microglia displayed amoeboid morphologies with higher fibrillar Aβ internalization and lysosomal activity, and primary cells and *BV2* lines exhibited increased uptake and degradation of Aβ. Altogether, these findings suggest that APOE–LILRB4 associated signaling may constrain microglial metabolic readiness through SHP‐2, NF‐κB, and STAT‐related pathways, and that *Lilrb4a* suppression favors a PPAR‐linked pro‐clearance state.

Our data also suggest compartment‐specific glial remodeling at parenchymal and vascular amyloid sites. *Lilrb4a* deficiency increased GFAP‐positive astrocyte coverage around parenchymal plaques and at CAA‐associated regions, and it also increased vascular smooth muscle coverage at CAA lesions (Figure 3I–M). In contrast, IBA1‐positive microglia did not increase perivascular association. A plausible explanation is that, at 4.5 months, CAA burden is below the threshold required for robust chemotactic recruitment or activation of perivascular microglia, whereas astrocytes can mount structural remodeling and redistribution in response to moderate vascular amyloid. This pattern is consistent with a compartment‐specific glial response in which microglia preferentially engage parenchymal plaques, whereas astrocytes show stronger structural engagement at vascular amyloid sites. Because *APOE4* is closely linked to the amyloid‐related imaging abnormalities (ARIA) risk during anti‐amyloid immunotherapy, these findings also raise a testable hypothesis that modulation of *APOE4*‐associated inhibitory signaling may influence vascular Aβ handling and neurovascular responses relevant to ARIA susceptibility. However, we did not directly assess neurovascular function or vascular integrity, nor did we examine anti‐Aβ antibody treatment or magnetic resonance imaging‐based edema or hemorrhage endpoints. Thus, any implication for ARIA remains speculative and will require dedicated vascular, longitudinal, and immunotherapy‐relevant studies.

Pharmacological and RNA‐targeting perturbations were used here primarily as mechanism‐oriented tools to probe pathway sufficiency and perturbability. PPAR‑γ agonism increased Aβ uptake and digestion in vitro without changing *Lilrb4a* transcript levels, indicating downstream or parallel enhancement of clearance (Figure 4F–K). In sorted microglia derived from *APOE3* and *APOE4* mice, PPAR‐γ inhibition abolished the gains in phagocytosis and degradation induced by ASO‐mediated *Lilrb4a* knockdown, supporting a functional requirement for PPAR‐γ signaling in this in vitro microglial context (Figure 4K–N). These findings suggest that the microglial clearance phenotype induced by *Lilrb4a* deficiency is not restricted to *APOE4* under the tested in vitro conditions. Although APOE4 associates more strongly with Lilrb4a, APOE3 may still engage the same inhibitory receptor axis to a lesser extent. Accordingly, both *APOE3‐* and *APOE4‐* derived microglia showed enhanced clearance‐related responses after *Lilrb4a* knockdown under the tested in vitro conditions, suggesting that the pathway is not exclusive to APOE4 even though APOE4 showed a stronger association with Lilrb4a in our Co‐IP experiments. Our study did not address the consequences of *APOE* deficiency or the potential involvement of *APOE2*, and these questions warrant further investigation. In microglia–neuron co‐cultures, PPAR‑γ treatment improved neuronal survival under inflammatory challenge (Figure 4O,P). In vivo, PPAR‑γ agonism reduced plaque burden, Aβ coverage, and dystrophic neurites, mirroring key aspects of the *Lilrb4a*‐deficient phenotype (Figure 5B–F). ASO‐mediated *Lilrb4a* suppression reproduced key effects of genetic knockout on plaques, CAA, and neuritic injury, providing proof‐of‐concept support for RNA‐based intervention in this model (Figure 7B–J). Taken together, these results support *Lilrb4a* suppression as a proof‐of‐concept intervention strategy in this model, while positioning PPAR‐γ modulation primarily as a mechanistic probe of the downstream pro‐clearance program rather than as a stand‐alone translational claim.

These findings suggest a mechanistic link between APOE4‐associated signaling and microglial dysfunction. *APOE4* has been linked to impaired microglial compaction and heightened neuritic injury; our data suggest that engagement of Lilrb4a biases downstream signaling toward a proinflammatory, metabolically constrained state. Disrupting this axis—via *Lilrb4a* suppression and/or enforcing a PPAR‐γ‐dominant program—rebiases microglia toward efficient plaque‐associated debris removal while engaging astrocytic support at vascular sites.

Limitations should be acknowledged. The study focuses on amyloid‐driven pathology; effects on tauopathy and cognition will require longer or earlier interventions and tau models. In addition, although our Co‐IP results support APOE–LILRB4 association, they do not on their own establish direct receptor–ligand binding or relative binding affinity. Direct in vivo microglia‐specific target engagement was not measured in ASO‐treated 5EL mice or in pioglitazone‐treated mice, which limits mechanistic and translational inference from these intervention arms. Microglia‐targeted specificity of PPAR‑γ agonism remains to be optimized due to potential systemic actions. Although pathological progression in 5EL mice is rapid, the timing of the ASO and PPAR‐γ agonist interventions remains limited to a relatively early pathological window and does not address treatment of established disease, and long‐term monitoring is lacking. In addition, prior clinical studies of PPAR‑γ agonists in AD have shown limited efficacy, underscoring the need for more precise target‐engagement, biodistribution, and cell‐type‐specific intervention strategies. Conditional, microglia‐specific *Lilrb4a* deletion and time‐course analyses across CAA progression would strengthen causal inference for perivascular recruitment thresholds. Finally, comprehensive safety and pharmacokinetics for ASO approaches are needed to inform translational development.

In conclusion, our data support Lilrb4a as an APOE4‐associated microglial checkpoint candidate linked to SHP‐2/NF‐κB/JAK–STAT‐related signaling changes and a PPAR‐linked pro‐clearance program. Its suppression—alone or in combination with PPAR‑γ activation—enhances microglial phagocytosis and lysosomal degradation, increases astrocytic engagement with plaques and CAA, reduces neuritic injury, and alleviates vascular amyloid without altering APP processing. These findings support further investigation of *Lilrb4a* and associated NF‐κB/PPAR‐linked signaling in *APOE4*‐associated amyloid contexts.

## 4 Experimental Section

### 4.1 Animals and Breeding

*Lilrb4a* knockout (*Lilrb4a−/−*, L4KO) mice were obtained from Saiye Biotechnology (China); 5xFAD and human *APOE4* knock‐in (*APOE4+/+*) mice were from the University of Science and Technology of China. 5xFAD mice were crossed with *APOE4* knock‐in mice, and L4KO mice were crossed with *APOE4* knock‐in mice to generate 5xFAD/*APOE4+/−* and *APOE4+/−*/*Lilrb4a+/+*. Subsequent interbreeding yielded final strains: 5xFAD/*APOE4+/+*/*Lilrb4a−/−* (5ELKO), 5xFAD/*APOE4+/+*/*Lilrb4a+/+* (5EL), and *APOE4+/+*/*Lilrb4a−/−* (ELKO), along with heterozygotes. Mice were maintained under specific pathogen‐free conditions (22°C; 12 h light/dark cycle; ad libitum food and water). Experiments were approved by the Ethics Committee of Chongqing Medical University and followed ARRIVE guidelines (approval number: IACUC‐CQMU‐2024‐0710). All animals were sampled at 4.5 months of age.

### 4.2 Perfusion and Tissue Preparation

Anesthesia was induced by intraperitoneal injection of 5% pentobarbital sodium (100 µL/10g). Blood was collected via the cardiac apex, centrifuged at 5500 × g (10 min, 4°C) to obtain plasma. Mice were perfused with ice‐cold phosphate‐buffered saline(PBS) (11 mL/min, 3 min) until the liver blanched. Brains were removed; the left hemisphere was fixed in 4% paraformaldehyde (PFA, 4°C, overnight), cryoprotected in 25% then 30% sucrose, sectioned (50 µm), and stored in cryoprotectant at −20°C. The right hemisphere was dissected into anatomical regions and snap‐frozen at −80°C.

### 4.3 ASO and PPAR‐γ Agonist Administration

Custom ASO targeting *Lilrb4a* mRNA (ASO‐L4a; 5'‐TAGTAACACTTATATATCCC‐3') was synthesized by SicaGene Biotechnology. Under isoflurane anesthesia, 2.5‐month‐old 5EL mice received unilateral intracerebroventricular injection (5 µL, 0.5 µL/min, 30 nmol) using a stereotaxic frame (RWD) and Hamilton syringe; coordinates: M/L −1.0 mm, A/P −0.3 mm, D/V −2.5 mm. Needle dwell time was 5 min post‐injection before withdrawal; incisions were sutured and disinfected. PPAR‐γ agonist pioglitazone (MCE, HY‐13956) was given by gavage (80 mg/kg, 5×/week) from 2.5 months; controls received vehicle.

### 4.4 Behavioral Experiments

EL and ELKO mice underwent behavioral testing at 4.5 months of age (*n* = 10 per group; 5 males and 5 females). 5EL and 5ELKO mice underwent behavioral testing at 4.5‐5.0 months of age (*n* = 10 per group; 5 males and 5 females). Mice were habituated to the testing room and apparatuses one day before testing. Behavioral testing was conducted in a quiet room during the light phase. EPM: Apparatuses were cleaned with 75% ethanol between animals, and testing was conducted under consistent environmental conditions. OFT: At the start of the experiment, the camera was activated, and the mouse was placed in the center of a square box. The mouse was allowed to explore freely for 10 min, during which absolute silence was maintained to prevent any disturbance. After the 10‐min period, the mouse was removed. Y‐Maze: At the start of the experiment, the camera was activated, and the mouse was placed at the far end of arm A, with its head facing the center of the maze. The mouse was allowed to explore freely for 8 min, while absolute silence was maintained to prevent disturbance. After an 8‐min period, the mouse was removed. NOR: The test consisted of two phases. On Day 1, mice were allowed to explore two identical objects fixed to the chamber floor for 10 min. On Day 2, one familiar object was replaced by a novel object of similar size but different shape, and mice explored the arena for another 10 min. Time spent exploring each object was recorded, and the Preference Index was calculated as (Tn − Tf)/ (Tn + Tf) × 100%, where Tn and Tf represent time spent exploring the novel and familiar objects, respectively. Behavioral data were analyzed blinded to genotype.

### 4.5 Isolation of Primary Microglia From Neonatal Mice

Primary microglia were isolated from postnatal day 2 (P2) C57BL/6J and L4KO mice. Culture flasks were pre‐coated with 0.1 mg/mL poly‐L‐lysine (PLL; Sigma, P6282‐5MG). Fresh brain tissue was transferred to Medium M (45 mL DMEM, 5 mL FBS, 500 µL P/S), filtered through a 70 µm mesh, and then transferred to culture flasks for incubation. On day three, the medium was replaced with Medium M+ (45 mL DMEM, 5 mL FBS, 500 µL P/S, 50 µL GM‐CSF (5 µg /mL). Once the microglia had matured, cells were harvested for phagocytosis and degradation assays, as well as for Co‐immunoprecipitation (Co‐IP).

### 4.6 *BV2* Cell Culture

*BV2* cells (KCB, Cat# KCB 200770YJ; RRID: CVCL_0182) were used as an established in vitro model of murine microglia, as they retain key microglial features, including cytokine production and phagocytic activity, and are widely employed to study microglial inflammatory and clearance functions. Cells were maintained in high‐glucose DMEM (DMEM‐H) supplemented with 10% FBS and 1% penicillin–streptomycin, and the line was authenticated by the provider. Cells were harvested for phagocytosis and degradation assays.

### 4.7 Isolation of Primary Microglia From Adult Mice

CD11b‐positive microglia were isolated using a CD11b‐positive microglia isolation kit (RWD, K1306‐10) from 12‐month‐old *APOE3* and *APOE4* mice that had received intraperitoneal LPS injection (3 mg/kg) 24 h earlier, as well as from 6.0‐month‐old 5EL and 5ELKO mice. Brain dissociation was performed according to the manufacturer's instructions using the RWD adult mouse/rat brain tissue gentle dissociation kit (DHABE‐5003). Briefly, the enzyme mix was freshly prepared and pre‐activated in a 37°C water bath before use. Mice were transcardially perfused for 3 min with ice‐cold PBS containing 1% GlutaMAX. Brains were then removed, and the entire cortical tissue was dissected and transferred, together with the enzyme mix, into RWD SCT‐100 tissue processing tubes. Tissue was cut into small pieces with scissors, and the tubes were mounted upside down onto the RWD DSC‐400 single‐cell suspension preparation system equipped with a heating sleeve. Samples were processed using the program M_ABrain_Heater_2. Cell strainers were pre‐wetted before filtration, and the resulting cell suspensions were filtered and centrifuged at 300 × g for 10 min. Pellets were transferred to 15 mL conical tubes, resuspended in the debris‐removal reagent provided in the kit, and carefully overlaid with pre‐chilled PBS. Samples were then centrifuged at 3000 × g for 10 min at 4°C (acceleration 5, deceleration 3).

After preparation of the single‐cell suspension, cells were counted and the suspension was adjusted to 1 × 10^8^ cells/mL. A 100 µL aliquot of cell suspension (1 × 10^7^ cells) was incubated with 10 µL of mouse CD11b biotin antibody for 10 min at 2°C –8°C. Cells were then washed with 1–2 mL of buffer and centrifuged at 500 × g for 5 min, and the supernatant was completely removed. The cell pellet was resuspended in 100 µL of buffer, followed by incubation with 10 µL of magnetic beads for 15 min at 2°C –8°C. After washing again with 1–2 mL of buffer and centrifugation at 500 × g for 5 min, the cell suspension was loaded onto a separation column placed in an appropriate magnetic field. The column was pre‐equilibrated with 4 mL of buffer before sample loading. Flow‐through was collected, and the column was washed 2–3 times with 2 mL of buffer to collect unlabeled cells. After the buffer had completely passed through, the column was removed from the magnetic field and placed over a new collection tube. Labeled CD11b‐positive cells were then eluted with 2 mL of buffer using the plunger supplied with the column. A small aliquot of the isolated cells was stained with an anti‐CD45/CD11b antibodies (BioLegend, 101208, 157606) and analyzed by flow cytometry to verify successful enrichment.

Sorted adult microglia were seeded onto collagen IV‐coated culture plates (2 µg/mL). When cells were plated on glass coverslips, the coverslips were first coated with poly‐*D*‐lysine and then with collagen IV. Cells were maintained in a defined serum‐free microglia growth medium consisting of DMEM/F12 supplemented with penicillin/streptomycin/glutamine, N‐acetyl cysteine, apo‐transferrin, sodium selenite, IL‐34 (100 ng/mL), TGF‐β2 (2 ng/mL), and cholesterol (1.5 µg/mL). Cultures were maintained at 37°C in 5% CO_2_ and allowed to recover overnight before downstream stimulation or functional assays. When longer‐term maintenance was required, 50% of the medium was replaced every two days. In addition, sorted primary microglia from 5EL and 5ELKO mice were allowed to stabilize after plating and were then treated for 24 h with either a PPAR‐γ agonist (MCE, HY‐13956, 20 µm) or a PPAR‐γ inhibitor (mCE, GW9662, 10 µm), followed by western blot analysis.

### 4.8 Phagocytosis and Degradation Assays

Primary microglia isolated from C57BL/6 and L4KO mice were used without any pretreatment and were directly subjected to phagocytosis or degradation assays. *BV2* cells were pretreated for 24 h with either a PPAR‐γ agonist (MCE, HY‐13956, 20 µm), FABP7 inhibitor (MCE, HY‐145990, 10 µm), PCK1 inhibitor (MCE, HY‐128923, 10 µm), APOA1 agonist (MCE, HY‐121698, 20 µm), APOA1 inhibitor (TargetMol, T35633, 20 µm), or DMSO as a vehicle control.

For phagocytosis assays in sorted primary microglia from *APOE3* and *APOE4* mice, cells were treated with ASO‐L4a or control ASO (5 pmol per 1 × 10^4^ cells) for 36 h, followed by an additional 24 h in medium containing either a PPAR‐γ agonist (MCE, HY‐13956, 20 µm) or a PPAR‐γ inhibitor (MCE, GW9662, 10 µm). Cells were then switched to medium containing LPS (1 µg/mL) for 6 h, followed by incubation with either pHrodo‐labeled Aβ for live‐cell imaging using the Incucyte S3 system (Sartorius) or 200 nm FAM‐labeled Aβ oligomers for flow cytometric analysis using a SONY SA3800 cell analyzer. Phagocytosis was assessed after 1.5 h of substrate internalization.

For degradation assays, primary microglia from C57BL/6 and L4KO mice were incubated directly with substrate for 3 h to allow internalization, after which the medium was replaced with fresh medium lacking additional substrate, and degradation was assessed after a further 24 h. For degradation assays in sorted primary microglia from *APOE3* and *APOE4* mice, cells were first incubated with substrate for 3 h to allow internalization, after which the medium was replaced with fresh medium containing the indicated treatments, including ASO‐L4a or control ASO together with either a PPAR‐γ agonist or a PPAR‐γ inhibitor. Degradation efficiency was then assessed after an additional 24 h. *BV2* cells were processed in the same manner after the indicated pretreatments.

For live‐cell imaging experiments, images were acquired beginning at 0 h, with four random fields per well captured every 30 min. Images were analyzed using ImageJ. Flow cytometry data were analyzed using FlowJo.

### 4.9 Microglia‐Neuron Co‐Culture

The method for microglia extraction followed the previously described protocol. Cells were centrifuged to form a pellet, washed, and plated onto 24‐well tissue culture plates containing glass coverslips coated with Geltrex at a density of 50 000 cells per well in Neurobasal medium. The microglia were cultured for two days in microglia medium. Neurons were derived from embryonic day 17 WT mouse embryos. The dissection and isolation of the cortex followed the same methods as for microglial extraction, except that Neurobasal medium (Neurobasal + 1× B27 + 1× penicillin/streptomycin + 1× L‐glutamine) was used for washing. The dissociated neuronal suspension was washed with 1 mL of Neurobasal medium and resuspended in 400 µL of Neurobasal medium before plating. The prepared neurons were seeded on top of the glial cells at a density of 20 000 cells per well. In all cell culture experiments, only the central wells of the plate were utilized, with the peripheral wells filled with 1 mL of autoclaved double‐distilled water. After two weeks of co‐culture, cells were treated with either a PPAR‐γ agonist (20 µm) or DMSO as a vehicle control for 24 h, followed by LPS treatment (5 µg/mL) for 12 h. Cultures were then washed with PBS and processed for immunofluorescence staining. Cells were fixed with 4% paraformaldehyde (PFA) for 15 min, washed three times for 5 min each with PBS, permeabilized with 0.2% Triton X‐100 for 15 min, and blocked with 5% BSA for 1 h at room temperature. Neurons were then stained overnight at 4°C with microtubule‐associated protein 2 (MAP2) antibody (rabbit monoclonal, 1:500). The following day, cells were washed three times for 5 min each with PBS, incubated with the appropriate secondary antibody, and counterstained with 4′,6‐diamidino‐2‐phenylindole (DAPI, 1:500) to label nuclei. Images were acquired using a SpinSR10 spinning‐disk laser confocal microscope at 10× magnification. For each condition, three replicate wells were included, and three random fields were collected per well for analysis. MAP2‐positive area within each field was quantified using ImageJ, and measurements from the three fields were averaged to generate one value per well. Statistical analyses were conducted with individual well as the unit of analysis, and n represents the number of wells unless otherwise specified.

### 4.10 Enzyme‐Linked Immunosorbent Assay (ELISA)

Frozen cortical tissue (10 mg) from each individual mouse was homogenized in PBS. Each sample represented one mouse, and n refers to the number of mice. The homogenate was centrifuged at 15 000 rpm for 30 min at 4°C, and the supernatant was collected as the PBS‐soluble (salt‐soluble) fraction for ELISA. The pellet was then re‐homogenized in an equal volume of 5 m guanidine hydrochloride (pH 8.0) and incubated at room temperature with gentle rotation for one hour. After incubation, the sample was centrifuged again at 15 000 rpm for 30 min at 4°C, and the resulting supernatant was collected as the guanidine‐soluble (“insoluble”) fraction for ELISA. Aβ_42_ levels in the cortical tissue were quantified using an Aβ_42_ ELISA kit (Jiangsu Aidisheng Biological Technology Co.,Ltd, ADS‐1363H1) according to the manufacturer's instructions.

### 4.11 WB

For frozen brain tissue, approximately 10 mg of cortical tissue was collected from each sample, and pre‐chilled radioimmunoprecipitation assay (RIPA) buffer was added at a ratio of 50 µL per 1 mg of tissue. For cultured cells, 100 µL of RIPA buffer was added per 1 × 10^6^ cells. Protease and phosphatase inhibitors were added, and the mixture was thoroughly vortexed. Samples were then homogenized on ice using a handheld electronic homogenizer for approximately 10 s (ultrasonic treatment: 20% duty cycle, 1 s on/off for a total of 1 min). The samples were centrifuged at 12 000 rpm for 10 min at 4°C, and the supernatant was collected. Protein concentrations were determined using the Micro BCA Protein Assay Kit (Thermo Fisher, 23235). The samples were stored at −80°C for future analysis.

A total of 20 µg of protein was loaded onto 4%–12% SurePAGE gels and separated in 2‐(N‐morpholino)ethanesulfonic acid (MES) buffer. The proteins were then transferred to nitrocellulose membranes using the iBlot 2 system. The membranes were blocked with 5% milk in TBS containing 0.05% Tween‐20 (TBST) for 60 min at room temperature. Following blocking, the membranes were incubated overnight at 4°C with the following primary antibodies: anti‐LILRB4 (Thermo Fisher, rabbit polyclonal, 1:1000), anti‐APOE (CST, rabbit monoclonal, 1:1000), Flag‐Tag (Affinity, mouse monoclonal, 1:5000), anti‐APP‐C (Sigma, rabbit polyclonal, 1:1000), anti‐β‐Amyloid 1–16 (BioLegend, mouse monoclonal, 1:2000), anti‐pSHP2 (CST, rabbit monoclonal, 1:1000), anti‐SHP2 (CST, rabbit monoclonal, 1:1000), anti‐NF‐κB‐p65 (CST, rabbit monoclonal, 1:1000), anti‐pSTAT1 (CST, rabbit monoclonal, 1:1000), anti‐STAT1 (CST, rabbit monoclonal, 1:1000), anti‐pSTAT3 (CST, rabbit monoclonal, 1:2000), anti‐STAT3 (CST, rabbit monoclonal, 1:2000), anti‐PPAR‐γ (Proteintech, rabbit polyclonal, 1:1000), anti‐CYP2E1 (ABclonal, rabbit monoclonal, 1:1000), anti‐ARG1 (Proteintech, rabbit polyclonal, 1:1000), and anti‐TGF‐β (Proteintech, rabbit polyclonal, 1:1000). The next day, the membranes were incubated with the corresponding HRP‐conjugated secondary antibodies (Proteintech, 1:5000) for 1 h at room temperature. For tissue western blot analyses, each lane represented an individual mouse sample, and n refers to the number of mice. For primary‐cell western blot analyses, cells from five mice of the same genotype were pooled to generate one sample per genotype; these blots are presented as representative results, and no statistical analysis was performed. Immunoreactive bands were detected using an enhanced chemiluminescence (ECL) Western blot substrate (Zen BioScience) and visualized with a FUSION FX5 imaging system (Vilber). Band intensities were then quantified using ImageJ.

### 4.12 Co‐IP

*HEK‐293T* cells (ATCC, Cat# CRL‐3216; RRID: CVCL_0063) were used solely as a heterologous overexpression system for human plasmids in Co‐IP experiments. Because of their robust growth and high transfection efficiency, *HEK‐293T* cells are a standard platform for assessing protein–protein interactions, and the line was authenticated by the provider. Human *APOE3*, *APOE4*, and 3×Flag‐tagged human *LILRB4* plasmids were transiently expressed in HEK‐293T cells as indicated. In parallel, endogenous Co‐IP experiments were performed using primary microglia isolated from *APOE3* and *APOE4* mice as described above. For cell lysis, all cells were seeded in 6 cm dishes and subjected to the treatments described above (∼5 × 10^6^ cells per dish), and the required volume of pre‐cooled Cell lysis buffer (20 mm Tris‐HCl, 137 mm NaCl, 10% glycerol, 1% Triton X‐100, 2 mm EDTA) was calculated at a ratio of 300 µL. Protease and phosphatase inhibitors were added, and the mixture was thoroughly vortexed. Cells were subjected to 10 passes with a 1 mL syringe fitted with a long needle, followed by incubation on ice for 10 min to ensure complete protein lysis. The samples were centrifuged at 14 000 g for 10 min at 4°C, and the supernatant was collected. To the supernatant, the following antibodies were added: IgG (rabbit monoclonal, 1:200), APOE (rabbit monoclonal, 1:50), LILRB4 (rabbit monoclonal, 1:50), and Flag (rabbit monoclonal, 1:20), along with Protein A/G Magnetic Beads (MCE, HY‐K0202). The mixture was incubated at 4°C for 2 h, followed by four washes with 500 µL of wash buffer. Four hundred microliters of the sample were then added to the antibody‐bound magnetic beads, forming an “antigen‐antibody‐bead complex,” which was rotated on a mixing apparatus at 4°C for 2 h before collecting the magnetic beads. The beads were washed four additional times with 500 µL of wash buffer. Following the washes, 50 µL of 1 × SDS‐PAGE loading buffer was added to the beads, mixed thoroughly, and heated at 98°C for 8 min to elute the proteins. The beads were subsequently separated, and the supernatant was collected for SDS‐PAGE analysis.

### 4.13 Immunofluorescence (IF)

Sections of brain tissue were cut to a thickness of 50 µm using a cryostat and placed in cryopreservation solution and stored at ‐20°C. For staining, selected sections were transferred to 12‐well plates, equilibrated in TBS for 10 min, and washed 3 × 5 min in TBS. Sections were then permeabilized with 2 mL TBST (TBS + 0.25% Triton X‐100) per well for 20 min at room temperature on a shaker, followed by 3 × 5 min washes in TBS. X‐34 staining was performed using a pre‐prepared staining solution (40% ethanol, 60% TBS, 1:500 10N NaOH, and 1:5000 X‐34), and the samples were incubated at room temperature for 20 min. After staining, sections were rinsed 3 × 2 min in 40% ethanol/60% TBS and then 3 × 5 min in TBS. Blocking was performed for 1 h at room temperature in 5% BSA in TBST (2 mL per well). The sections were incubated overnight at 4°C with the following antibodies: Ionized calcium‐binding adapter molecule 1 (Iba1, Thermo Fisher, goat polyclonal, 1:300), APOE (CST, rabbit monoclonal, 1:500), HJ3.4 (1:500), Lysosome‐associated membrane protein 1 (LAMP1, rat monoclonal, Thermo Fisher, 1:500), Glial fibrillary acidic protein (GFAP, Abcam, goat monoclonal, 1:200), Alpha‐smooth muscle actin (α‐SMA, R&D, mouse monoclonal, 1:500), Platelet endothelial cell adhesion molecule‐1 (CD31, Proteintech, rabbit monoclonal, 1:200), and Cluster of differentiation 68 (CD68, Bio‐rad rat monoclonal, 1:200). The following day, the overnight‐incubated brain slices were washed three times with TBS for 5 min each. All subsequent procedures were performed in the dark. After incubation with the corresponding secondary antibodies in a 48‐well plate at room temperature for 1 h, the slices were washed three times with TBS and transferred to microscope slides, allowing them to air dry naturally. The slices were then rinsed three times with ddH2O and allowed to air dry again. Mounting was performed using ProLong Gold from Thermo Fisher.

X‐34 staining and X‐34/HJ3.4 staining were captured using an Olympus VS200 slide scanner, maintaining the same spatial dimensions of the brain slices. LILRB4, X‐34/LAMP1, X‐34/IBA1/APOE, X‐34/GFAP/CD31/α‐SMA and X‐34/CD68/IBA1/PSD95 staining were imaged using a SpinSR10 spinning disk laser confocal microscope. All images were acquired from the same spatial dimensions of the brain slices for whole‐brain imaging.

For quantification of plaque burden, CAA burden, and HJ3.4‐positive area (10×, z‐depth 20 µm, 5 optical sections), cortical regions of interest (ROIs) were manually outlined in ImageJ, and the percentage of positive signal area relative to the total ROI area was calculated. The quantification of plaque‐associated microglia (40×, z‐depth 20 µm, 10 optical sections), X‐34‐positive plaques were manually outlined in ImageJ, and the ROI was expanded threefold; the percentage of microglial positive signal within the expanded plaque‐associated region was then quantified. For analysis of plaque‐associated dystrophic neurites (LAMP1‐positive signal, 40×, z‐depth 20 µm, 10 optical sections), X‐34‐positive plaques were manually outlined in ImageJ, and the ROI was expanded twofold; the percentage of LAMP1‐positive signal within the expanded plaque‐associated region was then quantified. For analysis of intramicroglial APOE signal (60× oil, z‐depth 20 µm, 10 optical sections), X‐34‐positive plaques were manually outlined in ImageJ, and the ROI was expanded threefold, after which ROIs corresponding to microglia within this region were defined, and the proportion of APOE signal within microglia was quantified. For analysis of intramicroglial CD68, PSD95, and X‐34 signals (60× oil, z‐depth 20 µm, 80 optical sections), individual plaque‐associated microglia were reconstructed using the Surface module in Imaris, and the proportion of CD68, PSD95, and X‐34 signals within each reconstructed microglial cell was quantified. For microglial morphology analysis (60× oil, z‐depth 20 µm, 80 optical sections), individual plaque‐associated microglia were subjected to morphometric analysis using the Filaments module in Imaris. For analysis of CAA‐associated microglia, astrocytes, and α‐SMA (40×, z‐depth 20 µm, 10 optical sections), CAA lesions were manually outlined in ImageJ, and the ROI was expanded threefold; the percentage of microglial, astrocytic, and α‐SMA‐positive signals within the expanded CAA‐associated region was then quantified. CAA was defined as vascular amyloid deposition identified by the colocalization of X‐34 and CD31. For all high‐magnification analyses (40× and 60×), three random fields were selected from the cortical region above the subiculum in each mouse, and measurements from these three fields were averaged to generate one value per mouse. Thus, the experimental unit for all analyses was the individual mouse, and n represents the number of mice.

### 4.14 qPCR

Total RNA was extracted from frozen brain tissue or sorted microglia using pre‐cooled TRIzol reagent. For brain tissue, approximately 5 mg of frozen tissue was weighed and lysed in TRIzol at a tissue‐to‐reagent ratio of 1:100. For sorted microglia, freshly isolated cell pellets were directly lysed in TRIzol immediately after enrichment. One to two magnetic beads were added to the TRIzol buffer, which was then placed in a homogenizer and processed for 45 s, repeated three times. The mixture was removed and allowed to stand on ice until foaming subsided. To each tube, the appropriate volume of chloroform (TRIzol = 5:1) was added and mixed vigorously for 15 s. The samples were allowed to sit on ice for 3 min, then centrifuged at 12 000 rpm for 10 min at 4°C. The supernatant was collected into sterile, enzyme‐free centrifuge tubes. To the supernatant, the appropriate volume of isopropanol (TRIzol = 2:1) was added and mixed well. The samples were then centrifuged at 12 000 rpm for 10 min at 4°C, and the supernatant was discarded. The pellet was washed with 75% ethanol, followed by centrifugation at 12 000 rpm for 5 min at 4°C, and the supernatant was discarded. After drying for 5 min, 20–30 µL of DEPC‐treated water was added, and the mixture was gently mixed by pipetting. RNA concentration was measured using a NanoDrop spectrophotometer, and samples with an OD260/280 ratio between 1.8 and 2.1 were used.The samples were stored at −80°C. Primer sequences are provided in Table S1.

### 4.15 Bulk‐RNA Sequencing

Library preparation and sequencing were carried out by Novogene Co., Ltd. The sequencing samples were randomly selected from 5ELKO and 5EL mice, with all tissues taken from the thalamus. RNA extraction followed the same steps as described in the first section. The extracted RNA was quantified, with concentration >50 ng/µL, total RNA >500 ng, and OD260/280> 1.8. Samples meeting these criteria were used for library preparation and sequencing. The raw data were downloaded in FASTQ format. Quality control of the raw sequencing data was performed using FastQC (v0.11.9). Trimmomatic (v0.39) was used for trimming sequences to remove low‐quality bases, adapter sequences, and repetitive sequences. The parameters used were PE, ‐threads 50, TruSeq3‐PE‐2. fa: 2:30:10:2, LEADING:3, TRAILING:3, MINLEN:36, HEADCROP:15. HISAT2 (v2.2.1) was used to align the sequencing data to the mouse reference genome (GRCm38), generating output in BAM format. The BAM files were sorted using samtools (v1.16.1). Gene quantification was performed using featureCounts (v2.0.3) with the parameters ‐p, –countReadPairs, ‐T 32, ‐s 0, ‐t exon, ‐g gene_name. Differential expression analysis was performed using DESeq2 (v1.38.3). Because the samples were derived from heterogeneous bulk brain tissue and the cohort size was modest, transcriptomic findings were interpreted primarily at the pathway level, and exploratory gene‐level thresholds were used to guide downstream validation experiments. KEGG (Kyoto Encyclopedia of Genes and Genomes) pathway and Gene Set Enrichment Analysis (GSEA) were performed using the richR (v0.0.34) package.

The RNA‐seq data generated in this study have been deposited in the Gene Expression Omnibus (GEO) under accession number GSE324957. All other data supporting the findings of this study are available from the corresponding author upon reasonable request.

### 4.16 RNA Scope (*Pparg*)

RNA in situ hybridization was performed using the PinpoRNA RNA in situ hybridization kit (Guangdong Pinbio Vision Biotechnology Co., Ltd., Cat# 190161‐B1) according to the manufacturer's instructions. Sections were first baked and fixed with PFA. Endogenous peroxidase activity was then blocked with Pretreatment Solution A, followed by boiling in Pretreatment Solution B to retrieve *Pparg* target sites. Gene‐specific probes were hybridized in probe hybridization buffer at 40°C for 2 h. After sufficient washing to remove unbound probes, signal amplification was performed using a nucleic acid hybridization‐based cascade amplification system. Sections were sequentially incubated with Reaction Solution 1, Reaction Solution 2, and Reaction Solution 3 at 40°C for 25, 15, and 15 min, respectively, with wash steps between each incubation. Fluorescent labeling was developed using a fluorogenic substrate through tyramide signal amplification (TSA). A probe targeting the Escherichia coli DapB gene was used as the negative control, and a probe targeting the mouse housekeeping gene Ppib was used as the positive control.

### 4.17 Statistical Analysis

Given that prior studies and our preliminary assessments indicate pronounced sex‐dependent differences in 5xFAD pathology, single‐factor models risk conflating sex effects with genotype. Therefore, one‐way ANOVA, which cannot accommodate sex × genotype interactions, was deemed unsuitable for these comparisons. To properly evaluate genotype effects while accounting for sex, 5EL and 5ELKO cohorts were analyzed using two‐way ANOVA (factors: sex and genotype) in Prism 8 (GraphPad Software), followed by post hoc multiple‐comparison tests where appropriate. All data are presented as mean ± SEM unless otherwise indicated. For imaging‐based analyses, multiple fields, plaques, or cells were quantified within each mouse and averaged to generate a single animal‐level value; thus, the mouse was used as the biological replicate for group comparisons. For co‐culture experiments, measurements from multiple fields within each well were averaged to generate one value per well, and the well was used as the experimental unit. Experiments involving a single factor were analyzed using unpaired two‐tailed Student's *t* tests for two‐group comparisons or one‐way ANOVA for comparisons involving three or more groups. Image acquisition and quantification were performed using identical analysis settings across groups and blinded to genotype and treatment. *p*‐value less than 0.05 (*p* <0.05) was considered statistically significant. Significance levels were indicated as follows: ^*^ *p* <0.05, ^**^ *p* <0.01, ^***^ *p* <0.001, ^****^ *p* <0.0001.

## Author Contributions

**Changxu Nie**, **Xin Tian**, **Li Jiang**, and **Chao Wang** conceived and designed the study. Changxu Nie and Chao Wang analyzed the data. Changxu Nie, **Ruixi Yang**, **Xiaotong Wang**, and **Ping Jia** performed most of the experiments, assisted by **Xueqi Zhang**, **Yaqi Dai**, **Xue Bai**, **Sijia Duan**, and **Yufeng Li**. Changxu Nie and Chao Wang drafted the manuscript with input from all coauthors. All authors read and approved the final manuscript.

## Funding

This study was supported by grants from the National Natural Science Foundation of China (Grant ID: 82271470, C.W), Lingang Laboratory AD Special Project (Grant ID: LG‐GG‐202401‐ADA010100, C.W), Natural Science Foundation of Chongqing Municipal Bureau of Science and Technology (Grant ID: 2023NSCQ‐MSX3605, C.W) and the National Science Fund for Excellent Young Scholars (Overseas, C.W), Scientific and Technological Research Program of Chongqing Municipal Education Commission (KZD‐JI202400406, C.W).

## Conflicts of Interest

The authors declare no conflicts of interest.

## Ethics Statement

The animal experiments and procedures were approved by the Animal Ethics Committee of Chongqing Medical University and carried out in accordance with the Guide for the Care and Use of Laboratory Animals (the Guide) from the NIH.

## Supporting information




**Supporting File**: advs76105‐sup‐0001‐SuppMat.docx.

## Data Availability

The RNA‐seq data generated in this study have been deposited in the GEO under accession number GSE324957. Other data supporting the findings of this study are available from the corresponding author upon reasonable request.
