# Supplementary material for: Lilrb4a Suppression Reprograms Microglia to Mitigate APOE4‐Associated Amyloid Plaques and Cerebral Amyloid Angiopathy in Association With a PPAR‐Linked Pro‐Clearance State
Source: Adv Sci (Weinh). 2026 Jun 22:e24167. Online ahead of print. doi: 10.1002/advs.202524167 (PMC13336908; doi:10.1002/advs.202524167)
Supplement: Supplementary file 1 — Supporting File: advs76105‐sup‐0001‐SuppMat.docx. [file ADVS-9999-e24167-s001.docx]

Lilrb4a Suppression Reprograms Microglia to Mitigate APOE4-Associated Amyloid Plaques and Cerebral Amyloid Angiopathy in Association with a PPAR-Linked Pro-Clearance State

Changxu Nie^#1,2^, Ruixi Yang^#1^, Xiaotong Wang^#1^, Ping Jia^#1^, Xueqi Zhang^1^, Yaqi Dai^1^, Xue Bai^1^, Sijia Duan^1^, Yufeng Li^1^, Peng Zheng^3^ Xin Tian^4^*, Li Jiang^5^*, Chao Wang^6^*

^1^ Department of Psychiatry, The First Affiliated Hospital of Chongqing Medical University; Department of Neurobiology, School of Basic Medical Sciences; Key Laboratory of Major Brain Disease and Aging Research (Ministry of Education), Chongqing Medical University, Chongqing, China.

^2^ Graduate School of Guangzhou Medical University; Guangzhou National Laboratory, Guangdong Province, China.

^3^ Department of Neurology, The First Affiliated Hospital of Chongqing Medical University；Jinfeng Laboratory, Chongqing, China.

^4^ Department of Geriatrics, Laboratory of Research and Translation for Geriatric Diseases, The First Affiliated Hospital of Chongqing Medical University; Department of Neurology, The First Affiliated Hospital of Chongqing Medical University, Chongqing Key Laboratory of Major Neurological and Mental Disorders; Key Laboratory of Major Brain Disease and Aging Research (Ministry of Education), Chongqing Medical University, Chongqing, China. Electronic address: xintian@cqmu.edu.cn.

^5^ Department of Neurosurgery, the First Affiliated Hospital of Chongqing Medical University, Key Laboratory of Major Brain Disease and Aging Research (Ministry of Education), Chongqing Medical University, Chongqing, China. Electronic address: drjiangli2019@163.com.

^6^ Department of Psychiatry, The First Affiliated Hospital of Chongqing Medical University; Department of Neurobiology, School of Basic Medical Sciences; Key Laboratory of Major Brain Disease and Aging Research (Ministry of Education), Chongqing Medical University, Chongqing, China. Electronic address: chao.wang@cqmu.edu.cn

# These authors contributed equally to this work

* Corresponding author


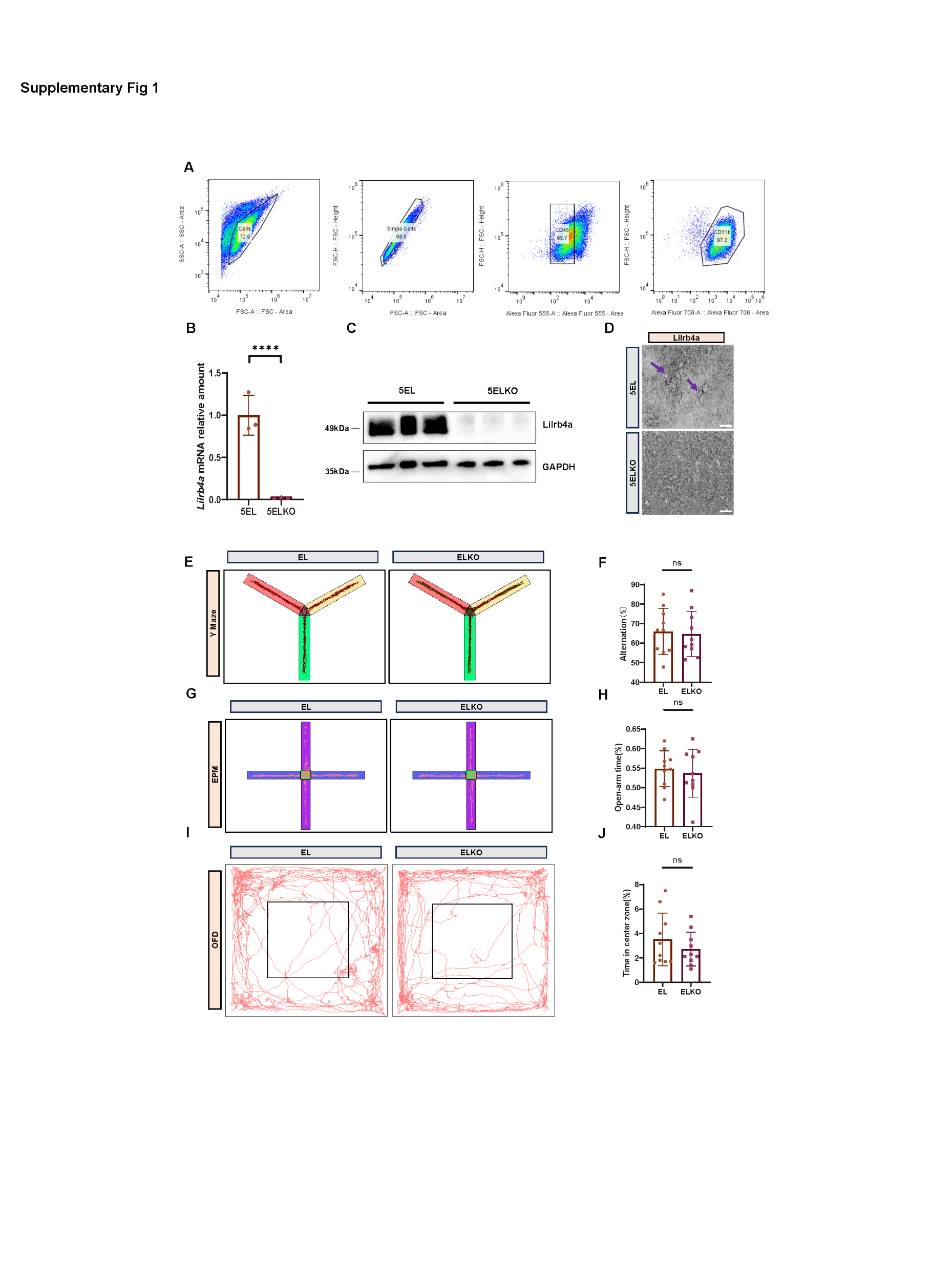


**Supplementary Figure 1. *Lilrb4a* knockout does not affect baseline behavior in mice.**

(A) Representative flow cytometry gating strategy used to verify the enrichment of magnetically isolated CD11b-positive microglia from adult mouse brains. Sequential gates were applied to select the main cell population, singlets, and the CD45/CD11b-positive fraction. Numbers indicate the percentage of cells within each gated population; (B) qPCR analysis of *Lilrb4a* mRNA expression in brain tissue from 5EL and 5ELKO mice (n = 3); (C) Representative immunoblot of Lilrb4a in brain lysates from 5EL and 5ELKO mice; (D) Representative Lilrb4a immunohistochemistry in brain sections from 5EL and 5ELKO mice, arrows indicate Lilrb4a-positive signal(40×, scale bar = 20 μm); (E, G, and I) Representative movement traces from the Y-maze (E), elevated plus maze (G), and open field test (I) in EL and ELKO mice; (F, H, and J) Quantification of spontaneous alternation in the Y-maze (F), time spent in the open arms in the elevated plus maze (H), and time spent in the center zone in the open field test (J) (n = 10, 5 males and 5 females); Data are presented as mean ± SEM. Statistical significance was determined by unpaired two-tailed Student’s t test (B, F, H, and J). *P < 0.05, **P < 0.01, ***P < 0.001, ****P < 0.0001; ns, not significant.


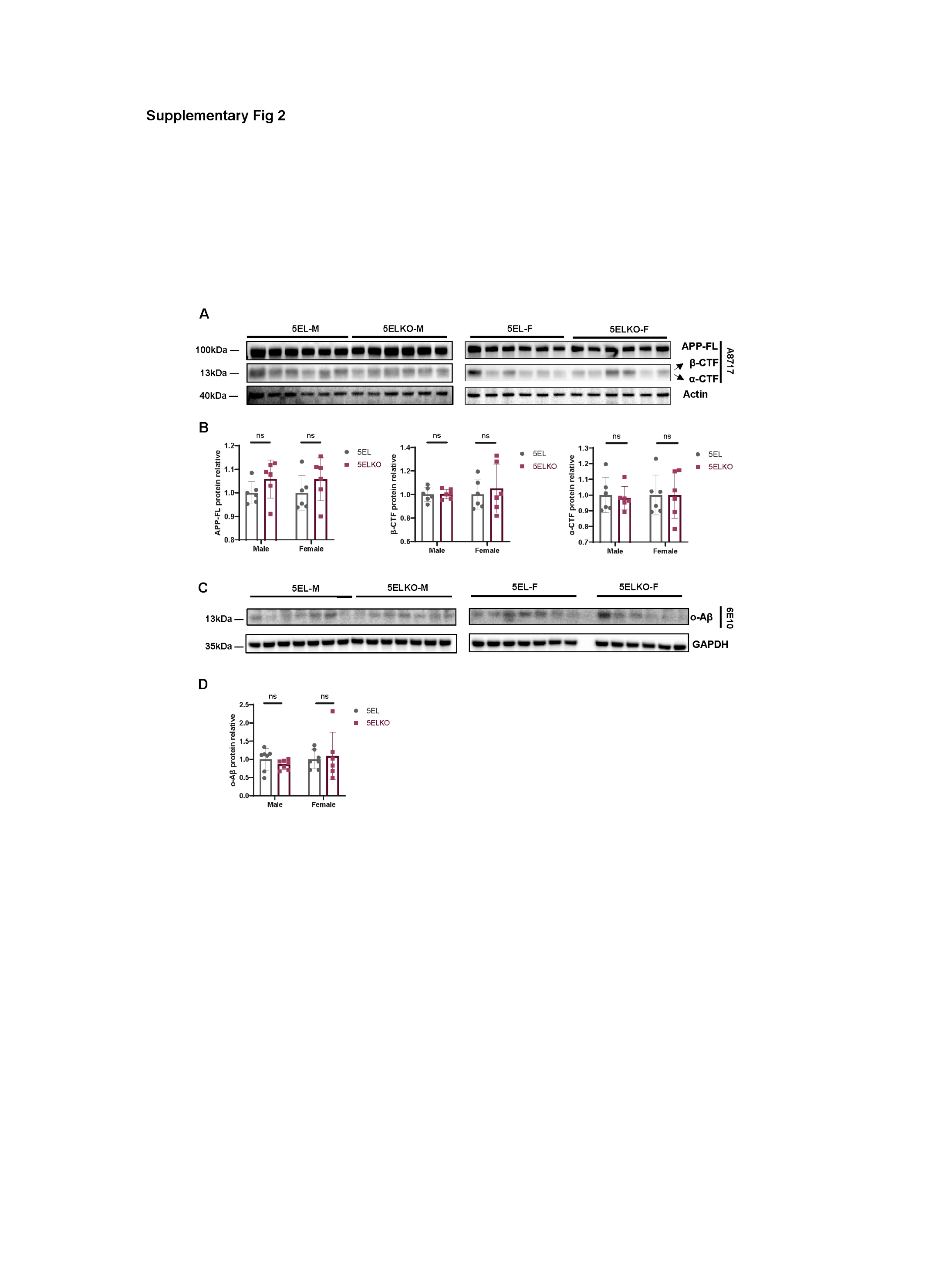


**Supplementary Figure 2.** ***Lilrb4a* deletion does not affect APP processing.**

(A) Representative immunoblot of APP processing in prefrontal cortex lysates from 5EL and 5ELKO mice using the A8717 antibody; (B) Quantification of APP full-length (APP-FL, left), β-CTF (middle), and α-CTF (right) protein levels (n = 6); (C) Representative immunoblot of oligomeric Aβ in prefrontal cortex lysates from 5EL and 5ELKO mice using the 6E10 antibody; (D) Quantification of oligomeric Aβ protein levels (n = 6-7). Data are presented as mean ± SEM. Statistical significance was determined by two-way ANOVA. ns, not significant.


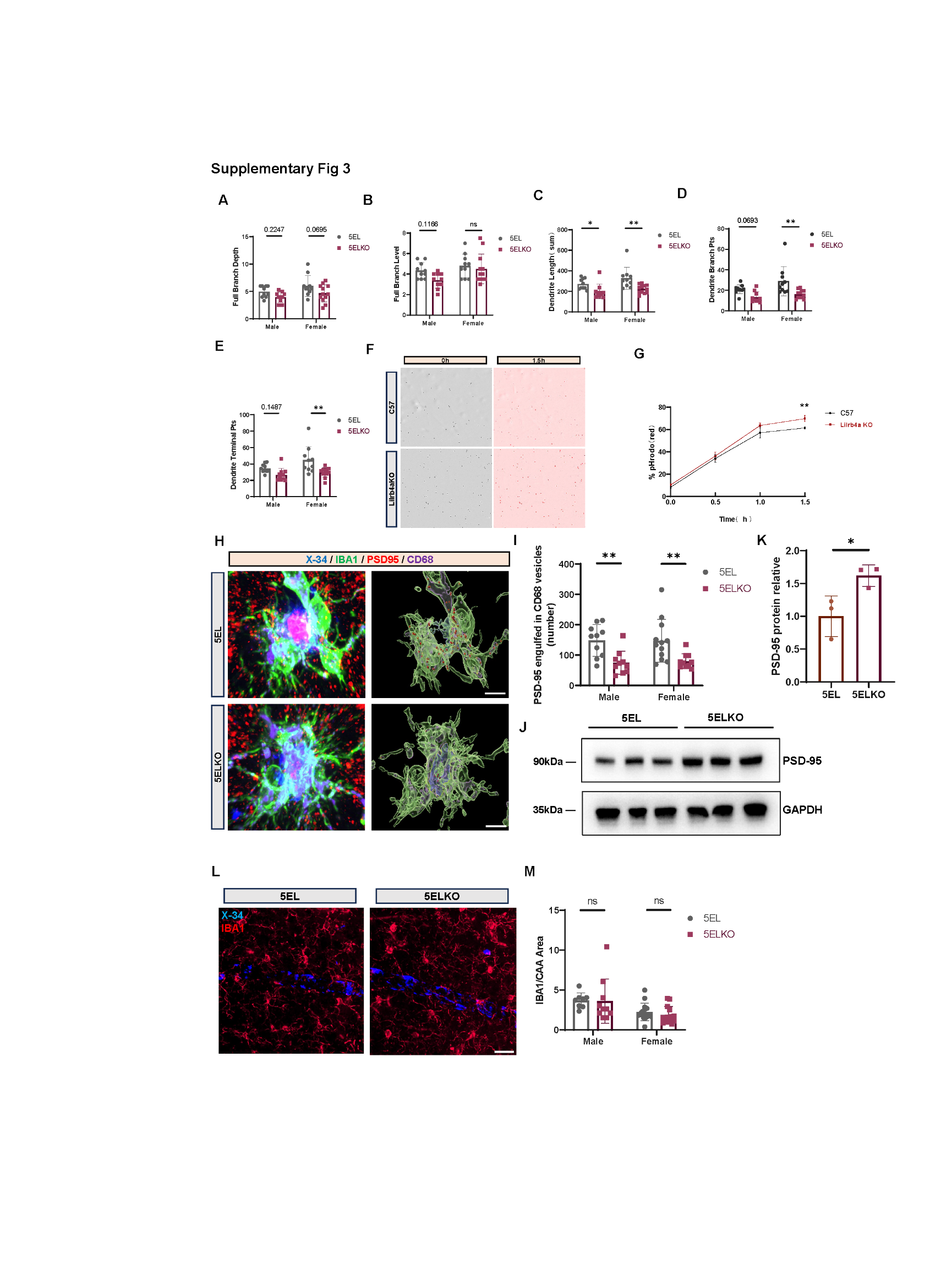


**Supplementary Figure 3. *Lilrb4a* regulates microglial morphology and clearance metrics; CAA‑associated microglia unchanged.**

(A–E) Quantification of full branch depth (A), full branch level (B), dendrite length (C), dendrite branch points (D), and dendrite terminal points (E) of plaque-associated microglia reconstructed using the Filament module in Imaris (n = 9–12); (F) Representative Incucyte images of pHrodo uptake in primary microglia from C57 and *Lilrb4a* KO mice at 0 h and 1.5 h; (G) Quantification of the phagocytosis rate, calculated as the percentage of pHrodo-positive area relative to total cell area; for each well, four random fields covering 1/12 of the well were acquired and averaged; three replicate wells were analyzed per group (n = 3); (H) Representative confocal images of X-34 (blue), IBA1 (green), PSD95 (red), and CD68 (magenta) staining in plaque-associated microglia from 5EL and 5ELKO mice; right panels show 3D reconstructions of microglia and engulfed material (60× oil, scale bar = 5 μm); (I) Quantification of PSD95 signal engulfed within CD68-positive vesicles in plaque-associated microglia (n = 9–12); (J) Representative immunoblot of PSD95 in cortical lysates from 5EL and 5ELKO mice; (K) Quantification of PSD95 protein levels in cortical tissue (n = 3); (L) Representative confocal images of X-34 (blue) and IBA1 (red) staining in CAA lesions from 5EL and 5ELKO mice (40×, scale bar = 10 μm); (M) Quantification of microglial coverage around CAA, expressed as the IBA1/CAA area ratio (n = 9–11). Data are presented as mean ± SEM. Statistical significance was determined by two-way ANOVA (A–E, I, and M), unpaired two-tailed Student’s *t* test (K), or two-way repeated-measures ANOVA (G), as appropriate. ns, not significant, *P < 0.05, **P < 0.01.


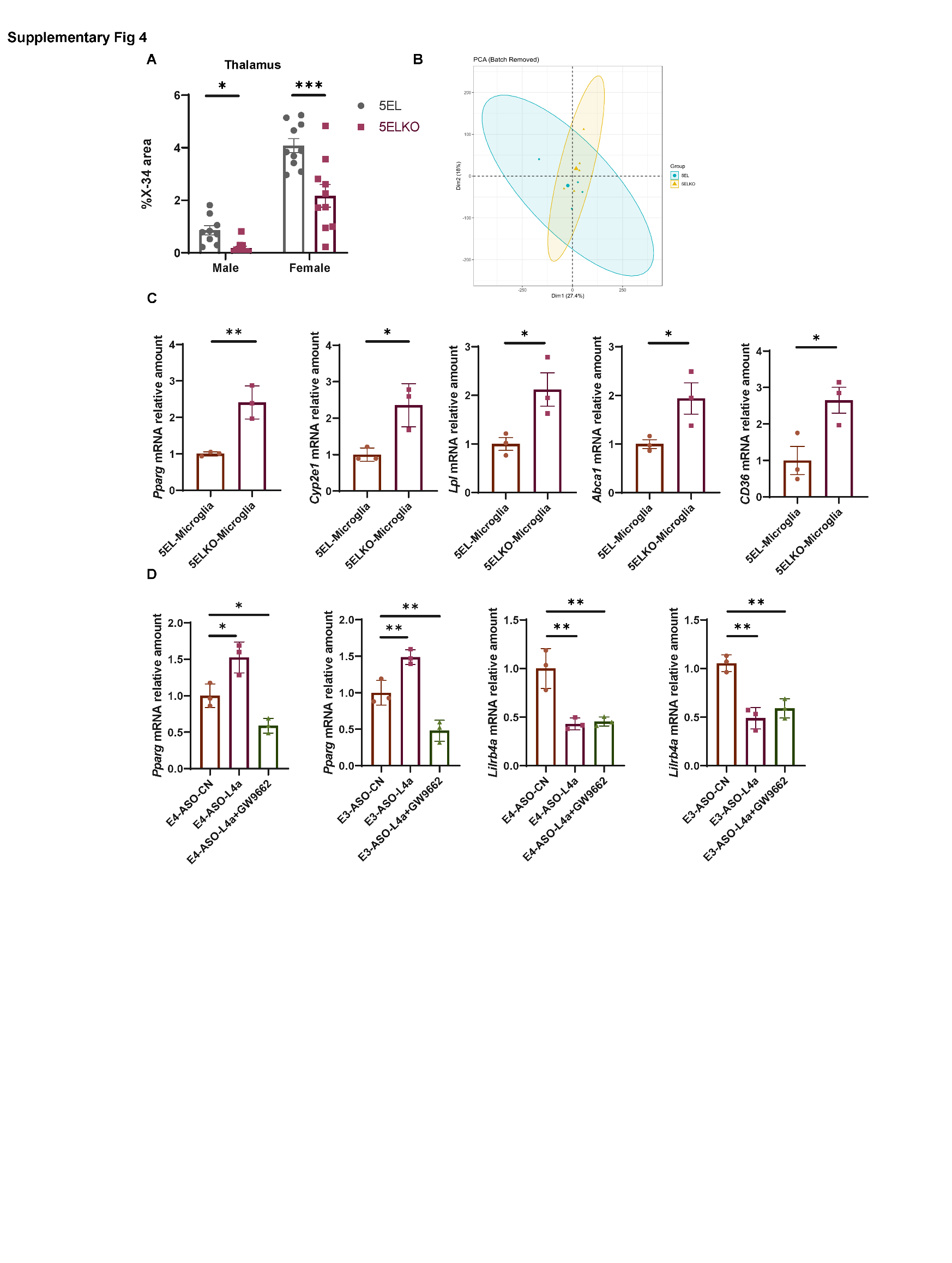


**Supplementary Figure 4. Thalamic plaque burden, principal component analysis, and microglial qPCR validation of the PPAR-related axis.**

(A) Quantification of X-34-positive area in the thalamus of male and female 5EL and 5ELKO mice (n=9-12), showing that the thalamus exhibited a pronounced pathological difference between genotypes and was therefore selected for bulk RNA-seq analysis; (B) Principal component analysis of bulk RNA-seq samples from 5EL and 5ELKO thalamic tissue; (C) qPCR analysis of *Pparg*, *Cyp2e1*, *Lpl*, *Abca1* and *Cd36* mRNA expression in sorted microglia isolated from 5EL and 5ELKO mice (n=3); (D) qPCR analysis of *Pparg* and *Lilrb4a* mRNA expression in sorted adult microglia isolated from LPS-treated APOE4 and APOE3 mice and subsequently treated *ex vivo* with control ASO, ASO-L4a, or ASO-L4a plus the PPAR-γ inhibitor GW9662 (n=3). Data are presented as mean ± SEM, statistical significance was determined by two-way ANOVA for (A), unpaired two-tailed Student’s *t* test for (C), and one-way ANOVA for (D). *P < 0.05, **P < 0.01, ***P < 0.001.


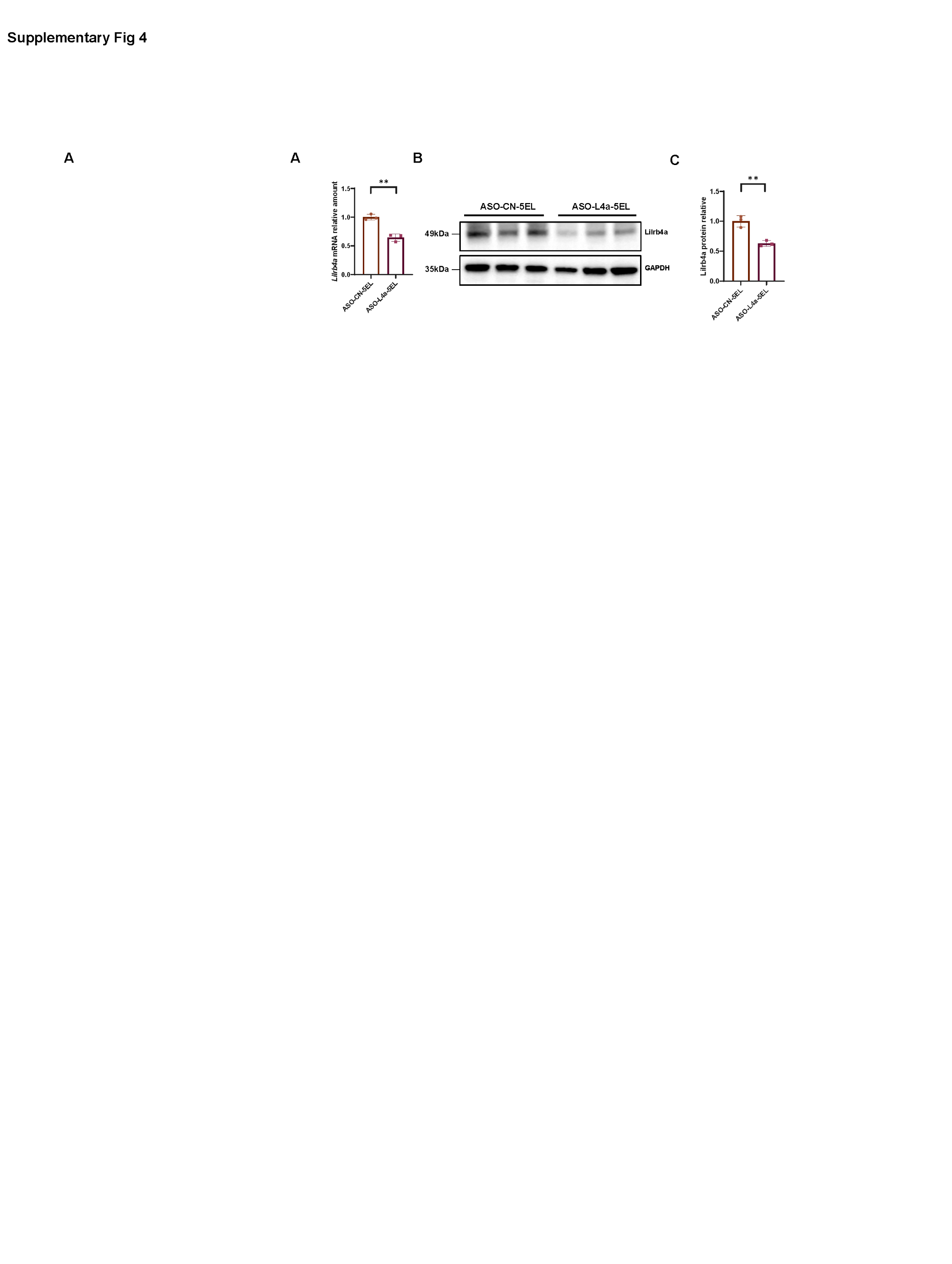


**Supplementary Figure 5.** **ASO targeting *Lilrb4a* reduces *Lilrb4a* mRNA and protein in 5EL cortex**

(A) qPCR analysis of *Lilrb4a* mRNA expression in cortical tissue from ASO-CN-5EL and ASO-L4a-5EL mice (n = 3); (B) Representative immunoblot of Lilrb4a in cortical lysates from ASO-CN-5EL and ASO-L4a-5EL mice; (C) Quantification of Lilrb4a protein levels in cortical tissue (n = 3). Data are presented as mean ± SEM. Statistical significance was determined by unpaired two-tailed Student’s *t* test (A and C). *P < 0.05, **P < 0.01.

| **Gene name** | **Primer sequence (5'-3')** |
| --- | --- |
| ***Lilrb4a*-F** | **CTGGATGCTGTTACTCCCAACC** |
| ***Lilrb4a*-R** | **TGGGTGTAGAGGACTGGTCCTT** |
| ***Apoa1*-F** | **GGCAGAGACTATGTGTCCCAGT** |
| ***Apoa1*-R** | **GCTGACTAACGGTTGAACCCAG** |
| ***Fabp7*-F** | **CAGTCAGGAAGGTGGCAAAGTG** |
| ***Fabp7*-R** | **GCTTGTCTCCATCCAACCGAAC** |
| ***Pck1*-F** | **GGCGATGACATTGCCTGGATGA** |
| ***Pck1*-R** | **TGTCTTCACTGAGGTGCCAGGA** |
| ***Plin2*-F** | **GACAGGATGGAGGAAAGACTGC** |
| ***Plin2*-R** | **GGTAGTCGTCACCACATCCTTC** |
| ***Apoa2*-F** | **CGGATATGCAGAGCCTGTTCAC** |
| ***Apoa2*-R** | **CTCGTGTGTCTTCTCAAAGTATGC** |
| ***Pparg*-F** | **GTACTGTCGGTTTCAGAAGTGCC** |
| ***Pparg*-R** | **ATCTCCGCCAACAGCTTCTCCT** |
| ***Cyp2e1*-F** | **AGGCTGTCAAGGAGGTGCTACT** |
| ***Cyp2e1*-R** | **AAAACCTCCGCACGTCCTTCCA** |
| ***Lpl*-F** | **GCGTAGCAGGAAGTCTGACCAA** |
| ***Lpl*-R** | **AGCGTCATCAGGAGAAAGGCGA** |
| ***CD36*-F** | **GGACATTGAGATTCTTTTCCTCTG** |
| ***CD36*-R** | **GCAAAGGCATTGGCTGGAAGAAC** |
| ***Abca1*-F** | **GGAGCCTTTGTGGAACTCTTCC** |
| ***Abca1*-R** | **CGCTCTCTTCAGCCACTTTGAG** |
| ***Gapdh*-F** | **CATCACTGCCACCCAGAAGACTG** |
| ***Gapdh*-R** | **ATGCCAGTGAGCTTCCCGTTCAG** |
| ***Actb*-F** | **CATTGCTGACAGGATGCAGAAGG** |
| ***Actb*-R** | **TGCTGGAAGGTGGACAGTGAGG** |

**Supplementary Table 1. All primer sequences for qPCR.**

| **Sample** | **Name** | **Q30** | **RIN** | **total reads** | **overall alignment rate (%)** | **unique mapping rate (%)** | **featureCounts assigned alignments (%)** |
| --- | --- | --- | --- | --- | --- | --- | --- |
| **5EL----149** | **5EL1** | **95.09** | **8.50** | **21338919** | **94.95** | **82.1** | **77.8** |
| **5EL----232** | **5EL2** | **94.84** | **8.40** | **25984291** | **94.85** | **79.89** | **76.6** |
| **5EL----313** | **5EL3** | **94.68** | **8.00** | **21367779** | **94.31** | **78.18** | **75.5** |
| **5EL----371** | **5EL4** | **95.15** | **8.2** | **21909641** | **94.82** | **80.86** | **77.6** |
| **5EL----374** | **5EL5** | **94.49** | **8.5** | **19876223** | **95.15** | **80.19** | **77.5** |
| **5EL+-+-379** | **5ELKO1** | **95.01** | **8.4** | **20831849** | **94.08** | **80** | **77.9** |
| **5EL+-+-408** | **5ELKO2** | **94.52** | **7.7** | **22692449** | **94.07** | **79.63** | **77.5** |
| **5EL+-+-412** | **5ELKO3** | **95.17** | **8.00** | **24908408** | **94.28** | **80.17** | **77.2** |
| **5EL+-+-419** | **5ELKO4** | **94.9** | **8.20** | **23621416** | **94.54** | **80.66** | **77.3** |
| **5EL+-+-424** | **5ELKO5** | **95.09** | **7.5** | **24437137** | **94.32** | **79.51** | **76.3** |

**Supplementary Table 2. Quality-control data for bulk RNA-seq.**
